# The COVID-19 Infodemic: Infodemiology Study Analyzing Stigmatizing Search Terms

**DOI:** 10.2196/22639

**Published:** 2020-11-16

**Authors:** Zhiwen Hu, Zhongliang Yang, Qi Li, An Zhang

**Affiliations:** 1 School of Computer and Information Engineering, Zhejiang Gongshang University Hangzhou China; 2 Beijing National Research Center for Information Science and Technology (BNRist), Tsinghua University Beijing China; 3 State Key Laboratory of Resources and Environmental Information System, Institute of Geographical Sciences and Natural Resources Research, Chinese Academy of Sciences Beijing China

**Keywords:** infodemiology, COVID-19, infodemic, social contagion, collective perceptual biases, collective behavioral propensities, social mobilization

## Abstract

**Background:**

In the context of the COVID-19 infodemic, the global profusion of monikers and hashtags for COVID-19 have found their way into daily communication and contributed to a backlash against China and the Chinese people.

**Objective:**

This study examines public engagement in crisis communication about COVID-19 during the early epidemic stage and the practical strategy of social mobilization to mitigate the infodemic.

**Methods:**

We retrieved the unbiased values of the top-ranked search phrases between December 30, 2019, and July 15, 2020, which normalized the anonymized, categorized, and aggregated samples from Google Search data. This study illustrates the most-searched terms, including the official COVID-19 terms, the stigmatized terms, and other controls, to measure the collective behavioral propensities to stigmatized terms and to explore the global reaction to the COVID-19 epidemic in the real world. We calculated the ratio of the cumulative number of COVID-19 cases to the regional population as the cumulative rate (*R*) of a specific country or territory and calculated the Gini coefficient (*G*) to measure the collective heterogeneity of crowd behavior.

**Results:**

People around the world are using stigmatizing terms on Google Search, and these terms were used earlier than the official names. Many stigmatized monikers against China (eg, “Wuhan pneumonia,” *G*=0.73; “Wuhan coronavirus,” *G*=0.60; “China pneumonia,” *G*=0.59; “China coronavirus,” *G*=0.52; “Chinese coronavirus,” *G*=0.50) had high collective heterogeneity of crowd behavior between December 30, 2019, and July 15, 2020, while the official terms “COVID-19” (*G*=0.44) and “SARS-CoV-2” (*G*=0.42) have not become de facto standard usages. Moreover, the pattern of high consistent usage was observed in 13 territories with low cumulative rates (*R*) between January 16 and July 15, 2020, out of 58 countries and territories that have reported confirmed cases of COVID-19. In the scientific literature, multifarious naming practices may have provoked unintended negative impacts by stigmatizing Chinese people. The World Health Organization; the United Nations Educational, Scientific and Cultural Organization; and the media initiated campaigns for fighting back against the COVID-19 infodemic with the same mission but in diverse voices.

**Conclusions:**

Infodemiological analysis can articulate the collective propensities to stigmatized monikers across search behaviors, which may reflect the collective sentiment of backlash against China and Chinese people in the real world. The full-fledged official terms are expected to fight back against the resilience of negative perceptual bias amid the COVID-19 epidemic. Such official naming efforts against the infodemic should be met with a fair share of identification in scientific conventions and sociocultural paradigms. As an integral component of preparedness, appropriate nomenclatures should be duly assigned to the newly identified coronavirus, and social mobilization in a uniform voice is a priority for combating the next infodemic.

## Introduction

### Background

The COVID-19 infodemic, associated with the COVID-19 outbreak, is getting some attention by researchers and policy makers [1,2]. On December 31, 2019, the novel coronavirus (2019-nCoV) disease was first reported from Wuhan City in China. With the spread of COVID-19, a massive information epidemic has undermined and disrupted global efforts to fight against COVID-19. However, the infodemic challenge does not receive enough attention in publications to fully understand it, and its unique risks have only begun to be explored [3]. The infodemic is partly characterized by a high information supply of information of variable quality, and a demand for timely and trustworthy information about 2019-nCoV [2,4-6].

On one hand, the global profusion of running headlines often inscribe fear, prejudice, disgust, and hostility into hashtags and monikers, branding discrimination and stoking panic [7-9]. Those monikers and morbid contents always combine with each other in the epicenter of an infodemic, wherein one sheds light on the social contagion of the other. The past few months have witnessed a growth of stigmatized monikers, which have found their way into daily communication and contributed to the backlash against China, Chinese people, and Asians in general [10-13]. Even worse, scientists frequently used similar monikers in the pandemic paper tsunami and exacerbated the situation [14,15].

On the other hand, individual perceptual bias could lead to insufficient or excessive information seeking, which further results in collective perceptual biases [16]. Pithy proper names are expected to be powerful in the ongoing campaign against the infodemic. As an integral component of preparedness, appropriate nomenclatures should be duly assigned to the newly identified coronavirus, which causes respiratory tract disease in humans and has had an impact on public health. So far, there are no universally accepted names, either for academic-industrial usage or consistency with international virus taxonomy.

To address such issues, we used available metadata to unfold the nature of the COVID-19 epidemic and the infodemic in this study [17].

### Study Objectives

An *inappropriate* official nomenclature might fuel the infodemics unconsciously. In recent years, humans have witnessed several outbreaks of infectious diseases caused by viruses, with common names given by stakeholders. Each round of naming practice is not always successful. As a case in point, some strongly held, but flawed, names such as “Middle Eastern Respiratory Syndrome” [18] and “Swine flu” were accused of unintentional social and negative economic impacts by stigmatizing certain industries or communities. “Swine flu,” an influenza strain that is known to have originated in pigs, resulted in financial damage to farmers, despite there being no evidence that it could be spread via pork consumption [19,20]. Since these incidents, in May 2015, the World Health Organization (WHO) released some naming conventions for the naming of new human diseases [21].

Infodemics long predate COVID-19 [22]. Unfortunately, with the spread of the COVID-19 epidemic, another massive infodemic has spread virally over the world with recurring episodes [23,24]. Previous evidence suggests that the internet, by its nature, could amplify and relay such infodemics swiftly worldwide, cause exaggerated panic, and progressively worsen stigmatization against people in the epicenter of an outbreak [23-26]. In the ongoing infodemic, Corona beer is being affected by the name’s similarity to the deadly coronavirus. In fact, the Mexican brand originated back in 1925 before the first strain of coronavirus was discovered and named. To address such challenges, the WHO declared this infodemic as the “2019-nCoV infodemic” on February 2, 2020 [4].

Based on a critical review, this study aims to take samples of the trillions of Google searches in connection with COVID-19 from December 30, 2019, to July 15, 2020, to address the following research issues:

- Did public engagement in the crisis communication reflect the collective sentiment of backlash against China and Chinese people in the real world? What were the global and geographical patterns of collective behavioral propensities to stigmatized monikers and the official terms?
- Were informed scientists well versed in the naming conventions to minimize unintentional negative impacts?
- What is the cohesive strategy of social mobilization to fight back against the COVID-19 infodemic?

## Methods

Infodemiology, a term coined by Eysenbach, is an emerging transdisciplinary area of research studying the epidemiology of information to address the pressing concerns of public health and policy decisions [2, 17,27,28]. The transdisciplinary nature of infodemiology can be found in Multimedia Appendix 1.

As of February 29, 2020, COVID-19 has spread to 60 countries and territories. Of these, the WHO published the number of cumulative cases in 54 member states on February 29, 2020, as well as Hong Kong, Macao, and Taiwan. We retrieved the cumulative cases of three nonmember states (Iceland, Azerbaijan, and Monaco) from their official websites. The corresponding total populations of 2019 come from the United Nations [29]. The cumulative rate *R* can be viewed as a ratio of the cumulative number of COVID-19 cases to the regional population: *R = i/p*, where *i* is the number of confirmed infections in a given country or territory and *p* is the national or regional population.

In this study, we retrieved metadata from three information sources: an electronic books corpus (Google Books Ngram Corpus [GBNC]), journals (Web of Science [WoS] and PubMed), and the internet (Google Trends Index [GTI]), and used them to facilitate subsequent analysis.

First, the GBNC provides a unique linguistic landscape that benefits from centuries of rich grammatical and lexical resources as well as cultural contexts [30]. Each taxonomic procedure often begins with a search through tomes for comparative morphologic variations to crystallize a pithy and appropriate neologism. For example, the earliest usage of *coronavirus* and *coronaviruses* could provide an insightful and compelling argument for the historical story and help us understand the essence of the name. The diachronic discourse of *coronavirus*, *coronaviruses*, *Coronaviridae,* and *Nidovirales* promises to articulate the unfolding chronological historical time scale from when these terms were first used [31].

Second, WoS and PubMed are publication databases with rich structural metadata. Scientometric analysis also promises to articulate the unfolding chronological picture of infodemiology. Under the umbrella of infodemiological scenarios, coupled with GBNC, scientometric analysis on diachronic discourses of pertinent keywords and phrases could reflect the historical milestones and the status quo in the field of human coronaviruses (HCoVs) research.

Third, the GTI part of this study commenced on December 30, 2019, and involved daily data collection worldwide until July 15, 2020, in 60 countries and territories that have reported confirmed cases of COVID-19 as of February 29, 2020. In the ongoing COVID-19 infodemic, stigmatized monikers against controls are ideal indicators of negative bias, and the unbiased and normalized GTIs were employed to determine their populational usages across various regions over time to characterize collective perceptual biases. GTI provides knowledge dissemination metrics for query incidences of relevant keywords and phrases, since about 63% of users on the internet use Google to search for ubiquitous information [32]. Three prominent strengths of GTI reflect the global reactions to major events in the real world: (1) vast data sets that include trillions of random searches; (2) unbiased sampling from the anonymized, categorized, and aggregated raw data; and (3) normalized indexes reconciling the dynamic nature of search volumes and the different population ratios in different regions. Therefore, the dynamic spatiotemporal pattern of GTI provides a unique lens into collective behavioral propensities of crowd behavior and demographic perception of a social contagion.

Intensive information seeking or avoidance choices may reinforce people’s cognition in both positive and negative ways, which is proactively rewarded by the feedback of the targeted information available [16]. In this study, a code scheme of subjective searches in daily communication was designed to fit three inclusion criteria: (1) top-ranked search interests, (2) formal and with complete spelling, (3) consistent with the global participants as much as possible. The collective behavioral propensities to the stigmatized terms were measured and compared with that of the control groups (the official terms and their counterparts). Herein, collective behavioral propensities to stigmatized terms directly represented the latent tendency to gather and interpret health care information available, which also reflected collective perceptual biases on preconceived judgements and social contagion [16,33-35].

To demonstrate the collective behavioral propensities, descriptive analysis and formal statistical analysis were carried out. For the descriptive analysis, the daily indexes of the relative search term volumes were separately mapped on a six-color rendering scale from 0 to 100. The earliest day of each terms debut was tracked and identified. Next, we introduced the Gini coefficient to measure the collective heterogeneity of crowd behavior. We denoted the search record of term *X* in the Google Trends data set as *X* = {*x*_1_, *x*_2_,...*x_n_*}, where *x_i_* represents the (normalized) search frequency of term *X* at the i-th time step. The mean value of *X* is: 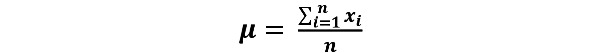


The Gini coefficient *G* for term *X* can then be calculated as follows:
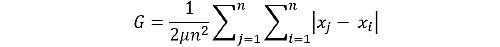


When the element values in *X* are equal, the Gini coefficient *G* takes the minimum value of 0, and when *x_i_*=0 for *i* = 1, ..., *n*–1 and *x_n_*=1, *G* will approach the maximum value of 1. The smaller the *G* is, the lower the collective heterogeneity of crowd behavior is and the higher the homogeneity of individual behaviors are. Conversely, it indicates that people are divided in the consistency of individual behaviors.

## Results

### The Enigmatic Nature of HCoVs Puts People on Edge

It is necessary to take a glimpse into the hierarchical Linnaean category of emerging coronaviruses [36,37]. As an international authoritative body, the WHO is responsible for naming new human infectious diseases. In 1966, the International Committee on Nomenclature of Viruses (ICNV) was established with the mission of introducing some degree of order and consistency into the naming of viruses. In 1973, the ICNV became the International Committee on Virus Taxonomy (ICTV), a global authority on the designation and naming of viruses. There are seven strains of HCoVs—HCoV-229E, HCoV-NL63, HCoV-OC43, HCoV-HKU1, severe acute respiratory syndrome–related coronavirus (SARS-CoV), Middle East respiratory syndrome–related coronavirus (MERS-CoV), and SARS-CoV-2—known to cause the common cold as well as more severe respiratory disease. Of those, HCoV-229E, HCoV-NL63, HCoV-OC43, and HCoV-HKU1 are routinely responsible for mild respiratory illnesses like the common cold but can cause severe infections in immunocompromised individuals, while the others have caused more severe diseases [38].

The diachronic discourse of *coronavirus* and *coronaviruses* in the English corpus unveils that there was a mild increase in the numbers of printed books dealing with HCoVs after the initial description of coronaviruses in 1968 [39-41]. The discovery of the novel strain had stimulated a new wave of research into coronavirus and the diseases it causes. Furthermore, meta-analysis results from WoS and PubMed indicated that the known knowledge remains off-limits in the field of combating emerging HCoVs [42]. The WHO declared the 2019-nCoV outbreak a Public Health Emergency of International Concern (PHEIC) on January 30, 2020. This is the sixth time the WHO has declared a PHEIC since the International Health Regulations (IHR) came into force in 2005. Before COVID-19, there have been five global health emergencies since such declaration was formalized: swine flu (2009), polio (2014), Ebola (2014 and 2019), and Zika (2016). The detailed descriptions of diachronic discourse analysis and scientometric analysis in this study can be found in Multimedia Appendix 2.

SARS-CoV-2 is the seventh identified coronavirus that can cause diseases of the respiratory tract via human-to-human transmission. It caused a mysterious pneumonia outbreak that is spreading far more quickly than the SARS-CoV and MERS-CoV diseases [1,43,44], even though the epicenter of the outbreak was locked down to curb the pandemic spread [42,45]. Presently, its underlying mechanism of clinical severity is yet to be determined, although many fatal cases have occurred [46].

On the one hand, the outbreaks of SARS-CoV, HCoV-HKU1, and SARS-CoV-2 were initially linked to China and lead people into the deep-rooted impression of China as an unsanitary entity. Chinese “wet markets” have been widely depicted as unsanitary hot spots for the transmission of zoonotic diseases [15,47-56]. Moreover, China is inevitably vulnerable to be accused of lax epidemiological control over HCoVs [57-63].

On the other hand, the enigmatic nature of HCoVs and the many unknowns about these epidemics have put people on edge. Information overload always follows closely behind the epidemics caused by HCoVs, especially in the age of the internet [64]. This enigmatic nature deepens people’s anxiety in a way that makes them respond to provocative online posts, whether intentional or not.

### Collective Behavioral Propensities in the Public

**Figure 1:**
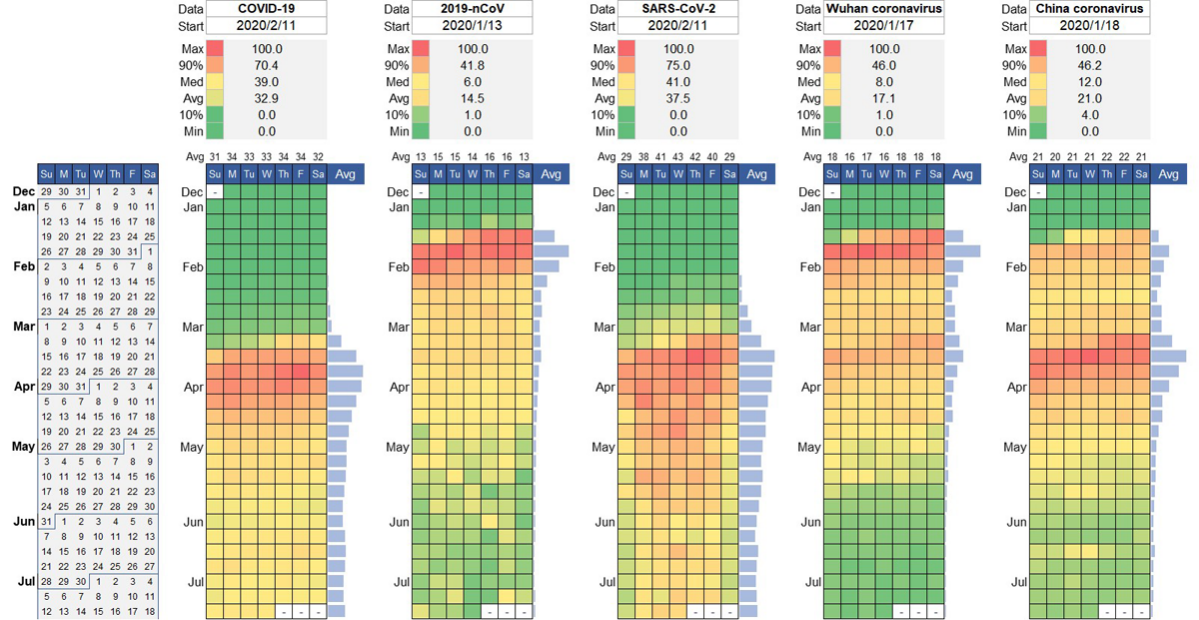
Calendar illustration on the relative search interest of the COVID-19 infodemic in the context of the COVID-19 epidemic (as of 15 July 2020; part 1).

**Figure 2:**
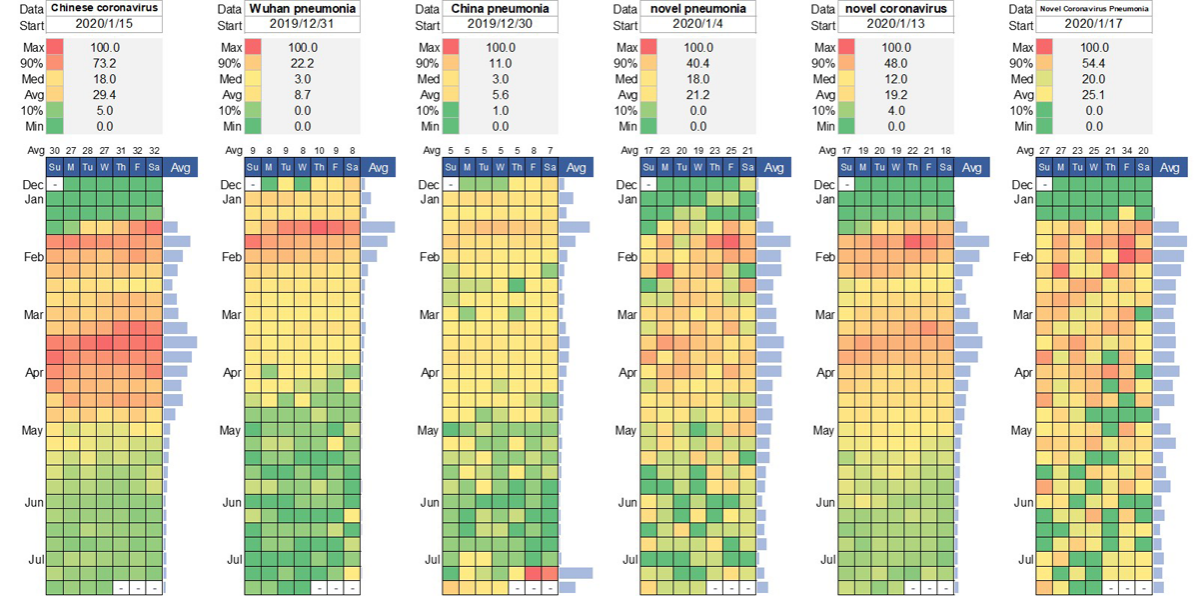
Calendar illustration on the relative search interest of the COVID-19 infodemic in the context of the COVID-19 epidemic (as of July 15, 2020; part 2).

We further examined what people were interested in and curious about with COVID-19. Google Trends showed the most-searched interest in the official terms (COVID-19, 2019-nCoV, SARS-CoV-2, and novel coronavirus pneumonia [NCP]), the stigmatized terms (Wuhan coronavirus, China coronavirus, Chinese coronavirus, Wuhan pneumonia, and China pneumonia), and other counterparts (novel pneumonia and novel coronavirus) from December 30, 2019, to July 15, 2020 (Figures 1 and 2). Those dynamic searches are indicators of collective behaviors across various regions over time. The detailed descriptions of the code scheme of multifarious naming practices can be found in Multimedia Appendix 3.

People around the world are divided in their own opinions on the internet and in daily communications. For the descriptive analysis, a striking feature was that some stigmatized monikers had comparatively high frequencies of collective usage. Being echoed by daily responses, the negative and lasting consequences pinpoint that those stigmatized names might have contributed to the recent backlash against China and Chinese people.

For the formal statistical analysis, first, the Gini coefficients of the stigmatized terms (eg, “Wuhan pneumonia,” *G*=0.73; “Wuhan coronavirus,” *G*=0.60; “China pneumonia,” *G*=0.59; “China coronavirus,” *G*=0.52; “Chinese coronavirus,” *G*=0.50) are significantly higher than those of the official terms (eg, “COVID-19,” *G*=0.44; “SARS-CoV-2,” *G*=0.42; “Novel Coronavirus Pneumonia,” *G*=0.46) and other controls (“novel pneumonia,” *G*=0.45 and “novel coronavirus,” *G*=0.49). This finding strongly indicates that the homogeneity of individual propensities to stigmatized monikers are lower than the official terms and the neutral names. The vulnerable population are highly susceptible to external negative sentiments. The 2019 novel coronavirus is thought to have originated in China, this misunderstanding may have led to the high usage of “Wuhan coronavirus,” “China coronavirus,” “Chinese coronavirus,” “Wuhan pneumonia,” “China pneumonia,” and other stigmatized monikers, even after July 2020. Second, after January 15, multifarious stigmatized monikers against Chinese people have prevailed in the public. “COVID-19” (*G*=0.44) took over from the premature name “2019-nCoV” (*G*=0.63), the latter finishing around February 28. Third, a notable pattern was observed after the announcements of the terms “COVID-19” and “SARS-CoV-2” (the collective usage of “SARS-CoV-2” has failed to match that of “COVID-19” in the public). The official terms “COVID-19” and “SARS-CoV-2” have not become the de facto standard usages. However, in the long run, the gradual increase in official names would be beneficial to correct ethnic stigmatization.

**Figure 3:**
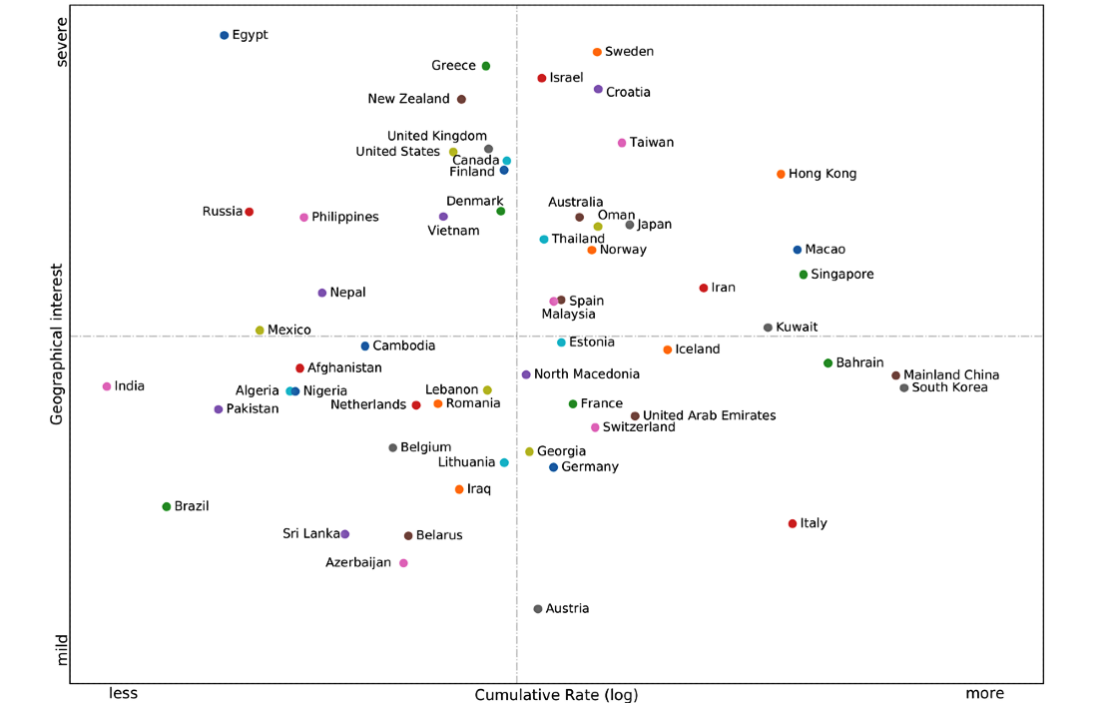
Four-quadrant diagram of the relationship between the geographical interest of stigmatized monikers and cumulative rate.

To further examine the demographical perceptions of collective behavioral propensities in the ongoing infodemic, we characterized the relationship between the geographical interest of stigmatized monikers and the cumulative rate of 58 countries and territories in which confirmed cases of COVID-19 have been reported. The results clearly unveil that people in Egypt, Greece, New Zealand, the United Kingdom, the United States, Canada, Finland, Russia, the Philippines, Denmark, Vietnam, Nepal, and Mexico prefer to use stigmatized monikers against Chinese people in comparison with other counterparts (Figure 3). As of February 29, 2020, up to 60 countries and territories have reported confirmed cases of COVID-19, including Taiwan, Iceland, Azerbaijan, and Monaco. There is no metadata available for San Marino and Monaco in Google Trends, but the geographical interest of stigmatized monikers against Chinese people in the other 58 territories was normalized by median volume to compare with each other. The cumulative rate is the ratio of the confirmed cases to the total populations in the countries or territories (Multimedia Appendix 4).

**Figure 4:**
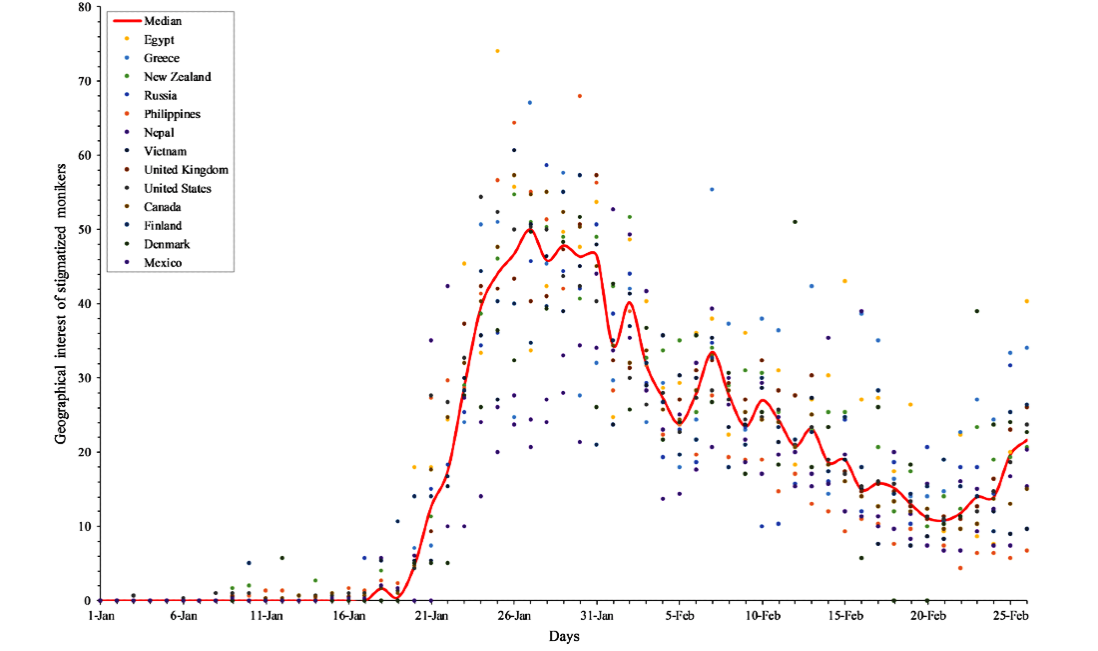
The dynamic interest of stigmatized monikers against China in territories with low cumulative rates.

To characterize the patterns behind such collective perceptual biases, we further scrutinized the geographical interest of stigmatized monikers against China in 13 territories with low cumulative rates over time (Figure 4). This illustration unveils the geographical interest of stigmatized monikers in 13 territories (Egypt, Greece, New Zealand, the United Kingdom, the United States, Canada, Finland, Russia, the Philippines, Denmark, Vietnam, Nepal, and Mexico) between January 1, 2020, and February 29, 2020. The median volumes of the corresponding search queries showed the trend of collective behavioral propensities over time. Comparatively, some stigmatized monikers against China saw high frequencies after January 16. The substantial pattern of the high consistent curve indicates that negative perceptual bias has been observed in the perception of the natural origin of COVID-19 in the public.

As a co-occurrence perceptual phenomena, the illustration could corroborate that people have used stigmatized monikers with high frequencies in these territories after January 16, 2020. People have a negative bias in their perception of COVID-19’s natural origin in these regions. Moreover, people hold negative perceptions of the authoritative responses in many countries [37,42,65,66]. The prognostic significance of our findings is that such approaches are expected to cause a psychological typhoon eye effect—a paradoxical phenomenon that the respondents closer to the epicenter of the pandemic appear to be the least concerned by the imminent risks—in the near future.

**Figure 5:**
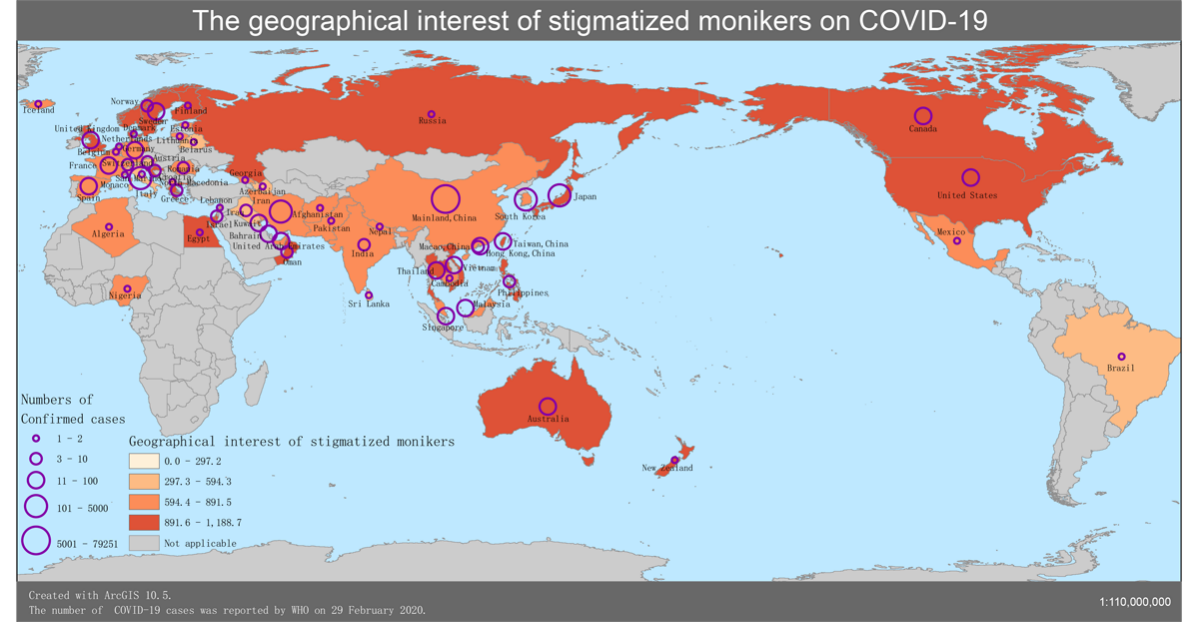
Geographical map of the COVID-19 infodemic (February 29, 2020). WHO: World Health Organization.

People hold negative perceptual bias in their perception of COVID-19’s natural origin in 58 countries and territories with low cumulative rates (*R*; Figure 5). The substantial pattern was observed on the geographical map of 58 countries and territories with low cumulative rates and negative perceptual bias in the perception of the natural origin of COVID-19 in the public. In the sociocultural setting with a relatively complex context beyond the epidemiological dimension, this panoramic map approach allowed us to better understand the prevalence and severity of the COVID-19 infodemic throughout the regions, comparatively. This finding reminds us that policy makers should learn from best practices to reduce deliberate infodemic risks, providing resources for knowledge and expertise in the academic sphere as well as in the public.

What are the plausible reasons behind such collective perceptual biases? Demographically, according to a Pew Research Center survey [67], a median of 40% of the surveyed countries had a positive view of China, compared with a median of 41% who had an unfavorable opinion. However, recognizing that COVID-19 has a potential public health impact, people are asking existential questions, making them vulnerable to the surfeit of information contagion from the outside world. When emerging cases were reported in their country, infodemics about the cause of the epidemic began, and nothing seemed certain or obviously right. As a case in point, the Bill & Melinda Gates Foundation, an American private foundation that has spent billions on global health care, have been suspiciously accused of manufacturing COVID-19 as biological warfare with the CIA to “wage economic war on China.” However, this was evidently not plausible. Such an infodemic campaign reminds us that authoritative organizations should work together with each other and cultivate a well-trained team of professionals to mediate infodemic risks.

From the beginning of the COVID-19 epidemic, people in most Asia-Pacific countries, “where many more name China as a top threat” [67], prioritized relations with China to jointly fight COVID-19, rather than use malicious discrimination against China. Regrettably, some individuals and media outlets have been committed to showing a negative image of China by promoting unfounded conspiracy theories such as a nonnatural origin of COVID-19 [68], a coronavirus that was made in China (even including desecration of the Chinese national flag), “China is the real Sick Man of Asia” [69], and China’s Chernobyl moment. Some instigators have made open apologies for spreading these rumors [14], but others are intent on whitewashing their words under the guise of freedom of speech.

Such voices do nothing but breed the pathogen of fear, panic, prejudice, disgust, xenophobia, and racism [9,14,70,71]. Undoubtedly, they have been met overwhelmingly with harsh criticism. On February 8, 2020, the *Lancet* published a statement in solidarity with Chinese professionals in combating the novel coronavirus outbreak and called for fighting against the infodemics [68,72]. Later, more public health scientists have endorsed this statement.

### Collective Perceptual Biases in the Scientific Community

Given that multifarious stigmatized monikers have become dominant in the public, what is being used in the scientific sphere? Admittedly, the plethora of papers on the pandemic have somewhat aggravated the collective perceptual biases, whether intentional or unintentional [73-75]. It is critical to have individuals who are well versed in naming conventions collaborate directly with researchers on a regular basis. Unfortunately, before the antidotes for the infodemic (ie, proper names) find their way into the public mind, debate on interim solutions has been ongoing (Multimedia Appendix 3).

On January 12, 2020, the WHO provisionally named the 2019 novel coronavirus disease “2019-nCoV.” China’s National Health Commission (CNHC) decided to temporarily call the disease “Novel Coronavirus Pneumonia” or “NCP” on February 7 (Figure 2). The CNHC’s official name has invoked intensive arguments outside as well as inside the scientific community. First, Chinese scientists are divided on that official name. Supporters say the descriptive name follows typical classification practices, whereas opponents claim that it could be easily misunderstood and abused to sow the seeds for panic. Second, the word *novel* is confusing because neither the disease nor the host can be used to reliably determine the virus’s novelty. Arguably, high mutation and gene recombination rates make this type of virus ideal for pathogen evolution [76]. Once viral mutation happens, it will no longer be *novel*.

Before that, the 2019 novel coronavirus was designated as “WH-Human-1 coronavirus” (“Wuhan-Human-1 coronavirus”) by a group of scientists in *Nature* on February 3, 2020 [77]. In the same vein, on February 11, another name, “HARS-CoV” (Han acute respiratory syndrome coronavirus) with *Han* standing for *Wuhan* in Chinese, was proposed in *The Lancet* (of note, some of the coauthors are members of the WHO IHR Emergency Committee) [15]. Obviously, such practices are against the naming principles of the WHO—geographic locations should be avoided in virus or disease names, and the proper names should be short and easy to pronounce [78]. Such names might provoke unintended negative impacts by stigmatizing Wuhan citizens and Chinese people. Those flawed notions take hold and should be duly corrected, as well as other similar paradigms (Table 1).

**Table 1:** Chronological list of published articles with multifarious proposed names.

Date (2020)	Article^a^	Proposed name
January 18	Cheng et al [79]	Wuhan coronavirus pneumonia
January 20	Parry [80]	China coronavirus
January 21	*Nature* [81]	Wuhan virus
January 22	Callaway and Cyranoski [82]	China coronavirus
January 22	Liu and Saif [83]	Wuhan coronavirus
January 23	Callaway and Cyranoski [84]	China virus
January 24	Mahase [85]	China coronavirus
January 28	Mahase [86]	China coronavirus
January 29	Parry [87]	China coronavirus
January 31	Callaway [88]	China coronavirus
January 31	Mahase [89]	China coronavirus
January 31	Bassetti et al [90]	Novel Chinese coronavirus
January 31	Ralph et al [47]	Wuhan virus
February 3	Wu et al [77]	WH-Human-1 Coronavirus^b^
February 4	Parry [91]	China coronavirus
February 5	Jiang et al [92]	PARS-CoV^c^
February 7	Cyranoski [93]	China coronavirus
February 11	Wang et al [15]	HARS-CoV^d^
February 11	Coronaviridae Study Group of the International Committee on Taxonomy of Viruses [38]	SARS-CoV-2
February 12	Zhou et al [94]	Wuhan novel coronavirus
February 14	Jiang and Shi [95]	TARS-CoV^e^
February 19	Jiang et al [96]	HCoV-19^f^
February 19	Goh et al [97]	Wuhan-2019-nCoV
February 19	Kooraki et al [98]	NCIP^g^
February 26	Xia et al [99]	NCP^h^

^a^The metadata of the articles were retrieved from PubMed as of February 26, 2020.

^b^WH-Human-1 Coronavirus: Wuhan-Human-1 coronavirus.

^c^PARS-CoV: pneumonia acute respiratory syndrome coronavirus.

^d^HARS-CoV: *Han* acute respiratory syndrome coronavirus.

^e^TARS-CoV: transmissible acute respiratory syndrome coronavirus.

^f^HCoV-19: human coronavirus 2019.

^g^NCIP: novel coronavirus-infected pneumonia.

^h^NCP: novel coronavirus pneumonia.

In response to such concerns, on February 11, 2020, the WHO officially renamed “2019-nCoV” as “COVID-19,” with *CO* meaning *corona*, *VI* for *virus*, *D* for *disease*, and *19* referring to 2019. This generic descriptive reassignment offers an overdue correction to those strongly held but flawed notions, with the hope of minimizing stigma. Coinciding with the WHO’s latest announcement, in a bioRxiv preprint [100], a new name “Severe Acute Respiratory Syndrome coronavirus 2” or “SARS-CoV-2” was penned by the Coronavirus Study Group of the International Committee on Taxonomy of Viruses (ICTV-CSG) on the same day. ICTV-CSG explains that this designation highlights the new strain’s similarity to SARS-CoV [38]. It is unclear whether this proposal name will be approved by the next plenary meeting of ICTV.

The WHO and some prominent virologists are far less skewed toward SARS-CoV-2, the nomenclature endorsed by ICTV-CSG [96,101]. Outside the academic-industrial sphere, people also argued against this proposed name. Although it seems natural for ICTV-CSG to add a numeral *2* behind “SARS-CoV” to signify their relation, many prominent scientists have scrambled to refute this claim. To the untrained eye, the hasty designation may mislead the public to perceive a more severe virus strain as a direct descendant of SARS-CoV, rather than just a close affinity for the causative agent of another major viral outbreak in China in 2002 and 2003. Before that, on February 5, 2020, Jiang and colleagues [92] proposed another name, “Pneumonia Acute Respiratory Syndrome Coronavirus” (“PARS-CoV”) in *Cellular & Molecular Immunology*. By the same token, this assignment also intends to retain equivalent terminology to SARS-CoV. Nonetheless, only 2 weeks later, without mention of their earlier similar formulations [92,95], they reintroduced the third name “HCoV-19” (“Human coronavirus 2019”) in *The Lancet* [96], objecting to the usage of SARS-CoV-2.

The looming worry is that the public are susceptible to SARS-CoV [25], which evokes the memory of the higher case fatality ratio. On February 9, 2020, Chen Huan-chun, a Chinese academician and virologist, made a public apology for mistakenly saying 2019-nCoV is SARS-CoV, which had struck a nerve and aroused great consternation in the Chinese public.

Mitigating infodemic risks by making informed and judicious choices is a catch-22 for authoritative organizations. It is necessary to punctuate heuristic cautions and continuous introspection of previous multifarious names [18,25,78,101], which is a requisite bedrock of such scientific efforts. Recently, global profusion of candidates has been discussed inside the scientific community, as well as on social media (eg, transmissible acute respiratory syndrome coronavirus [TARS-CoV] [95] and contagious acute respiratory syndrome coronavirus [CARS-CoV]). Whatever merits and demerits each term has, some of them should be fairly recognized with plausible reasons. Authorities should have an open mind to the modest introspections and rededications of such collective efforts. On February 22, 2020, CNHC officially renamed the temporary English name “NCP” as “COVID-19,” with the hope of standing with the WHO and further discouraging the use of stigmatized titles [102].

### Combating the COVID-19 Infodemic: Same Mission, Diverse Voices

**Figure 6:**
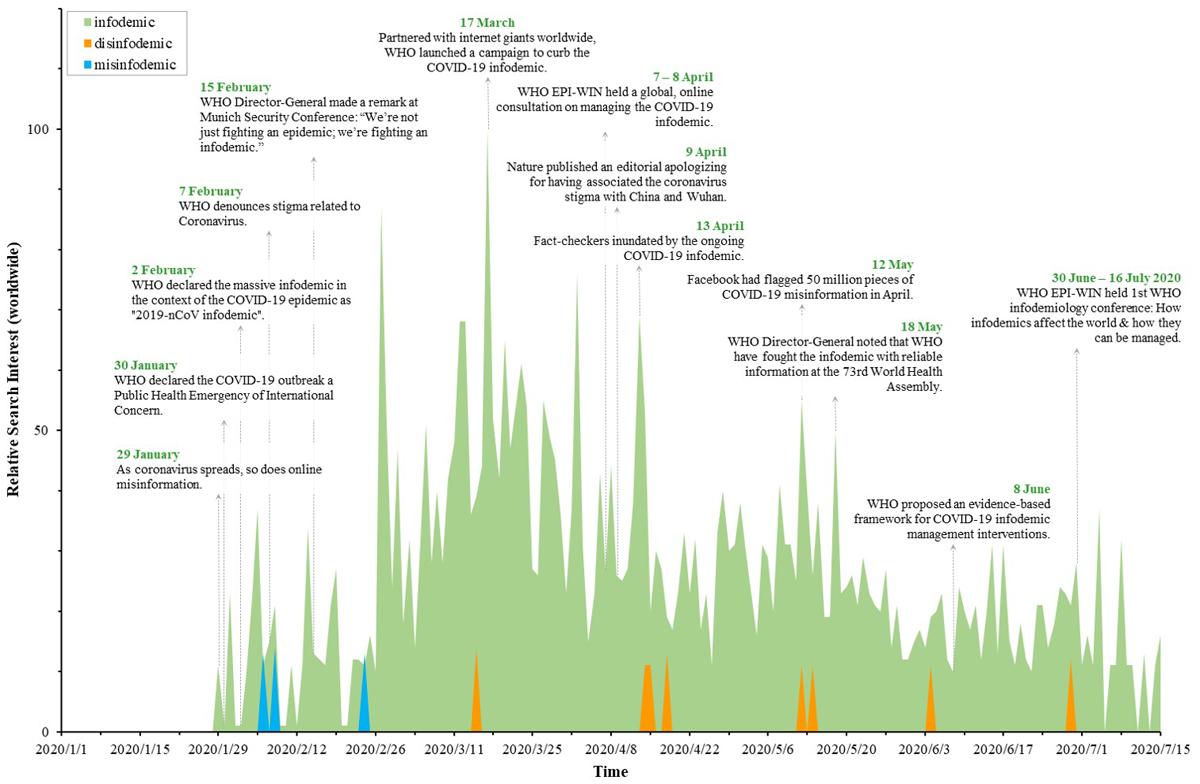
Illustration on the relative search interest of information contagion from around the world. EPI-WIN: Information Network for Epidemics; WHO: World Health Organization.

As the COVID-19 epidemic spreads, so does the information epidemic. The COVID-19 infodemic has introduced a new round of challenges for crisis communication, just as Dr Tedros Adhanom Ghebreyesus, the Director-General of the WHO, remarked at the Munich Security Conference on February 15, 2020: “We’re not just fighting an epidemic; we’re fighting an infodemic.” Infoveillance is an effective strategy against infodemics [34,35,103]. Unfortunately, with the same mission of corroborating reliable information and keeping people informed, different practitioners are upholding diverse voices in the campaigns against the ongoing information epidemic. As can be seen from Figure 6, Google Trends showed that the interest of the portmanteau words “infodemic,” “disinfodemic,” and “misinfodemic” from January 1, 2020, to July 15, 2020. The discourse system to address the present challenge is divided into three camps: the *infodemic* campaign endorsed by the WHO partnered with internet giants worldwide, the *disinfodemic* campaign backed by organizations led by the United Nations Educational, Scientific and Cultural Organization (UNESCO), and the *misinfodemic* campaign supported by other practitioners. Although the *infodemic* campaign is dominating the fray, most people are currently more interested in what is going on in the real world but are curious about what an infodemic is. The detailed descriptions of the code scheme of combating the COVID-19 infodemic in this study can be found in Multimedia Appendix 3.

In 2002, Eysenbach [27, 103] coined the portmanteau “infodemiology” (a novel transdisciplinary science to unravel the complex propagation patterns of misinformation and public health relevant information) along with the portmanteau “infoveillance” (a type of syndromic surveillance that uses online content). On February 2, 2020, the WHO adopted the term “infodemic” as an “overabundance of information – some accurate and some not – that makes it hard for people to find trustworthy sources and reliable guidance when they need it” [1]. In the aftermath of the online technical consultation on the COVID-19 infodemic [1], the WHO crystallized an evidence-based framework to underpin infodemic management interventions [1, 2, 104]. In the disinfodemic campaign, Posetti and Bontcheva [105] proposed the neologism “disinfodemic” (a blend of dis-, information, and epidemic) in the research-based policy briefs of UNESCO, considering its opposite of information [106]. A minority of researchers favor the term “misinfodemic” (a blend of mis-, information, and epidemic) in line with misinformation [107]. In contrast, “infodemic” is a more efficient portmanteau than “disinfodemic” or “misinfodemic” for communicative efficiency determined by shorter orthographic and phonetic length, according to Zipf’s [108,109] principle of least effort governing human lexicons.

On the other hand, it is critical to engage individuals to fully realize the damaging effect of infodemics and participate in the initiative of disproving COVID-19 myths. With social network giants worldwide, the WHO has been committed to curb the infodemics. For example, as part of the coordinated action, Facebook flagged around 50 million pieces of COVID-19–related content in April 2020. However, according to a Pew Research Center’s American News Pathways survey, about three in ten Americans still believe the viral conspiracy theories (COVID-19 was created in a lab and the COVID-19 outbreak was intentionally planned by people in power) [110]. Therefore, drawing on the lessons learned from the contextualized pollution of the media ecosystem, each one of us should contribute to the fight against both societal COVID-19 and information contagion in the most effective way.

## Discussion

### Principal Findings

With an emphasis on infodemiological analysis and meta-analysis on the COVID-19 epidemic and infodemic, we scrutinized the collective communication behaviors on the internet and pertinent usages in publications in sociocultural paradigms to uncover collective behavioral propensities and consequences.

First, psychologists often make claims about the relatedness between epidemics and panic based on qualitative evidence. The quantitative results reveal that people are invariably vulnerable to panic attacks during episodes of epidemics with an enigmatic nature. People around the world are divided in their favor of stigmatized monikers because of perceptual bias in the public and scientific communities. People in 13 (22%) out of 58 territories with low cumulative rates had negative behavioral propensities to stigmatized monikers in their daily communications. Perceptual bias in the perception of the natural origin of COVID-19 is part of the reason for specific regions, rather than the degree of infection in their territories.

Second, infodemics follow closely on the heels of every pathogen [5,6,23,24,37], branding discrimination and stoking panic. Official names would duly discourage the spread of regional stigmatization and racial discrimination, and reverse negative perceptual biases and collective behavioral propensities in public engagement.

Third, the coordinated campaign of fighting against the COVID-19 infodemic has called for an approachable uniform voice in line with the same mission, keeping lay audiences informed.

### Conclusion

With the benefit of hindsight provided by the Gini coefficient (*G*), the contextualized results indicate that many stigmatized monikers against China had a higher collective heterogeneity of crowd behavior than the official terms between December 30, 2019, and July 15, 2020. The prognostic significance of information seeking and avoidance is that infodemiological analysis could provide a hallmark reference to reframe extensible discussions on the COVID-19 epidemic and infodemic, as well as substantial patterns of the next infodemic.

At this critical moment, an epoch-making name is expected to be scientifically pithy and socially acceptable, with minimal unintentional negative impacts on nations, economies, and people. This is a positivist doctrine, not merely for naming a virus but for the vitality of science and the promotion of social progress. Obviously, some naming practices went awry, intentionally or not [14]. A learning lesson from the infodemic is the necessity of coming up with guidelines for the adoption of practical principles intended to enhance the possibility for the lessening of stigmatization and discrimination.

Technically, we now see collaborative efforts as a potential way to help strengthen and standardize ongoing international initiatives of the WHO and the ICTV [5,6]. Admittedly, understanding the way naming rules strengthen the integrity and quality of naming practices with the original mission remains nominal rather than substantial [18,25,78,101]. A *Nature* editorial remarked, “As well as naming the illness, the WHO was implicitly sending a reminder to those who had erroneously been associating the virus with Wuhan and with China in their news coverage — including *Nature*. That we did so was an error on our part, for which we take responsibility and apologize” [14]. As another precaution, the word *novel* was recommended by the WHO for “indicating a new pathogen of a previously known type, recognizing that this term will become obsolete if other new pathogens of that type are identified” [21]. However, stakeholders frequently reserve *novel* for indicating new types of viruses, lest this word fundamentally lose its impact without regular amendments.

## Appendix

Multimedia Appendix 1 The transdisciplinary nature of infodemiology.

Multimedia Appendix 2 Diachronic discourse analysis and scientometric analysis.

Multimedia Appendix 3 Code schemes.

Multimedia Appendix 4 Cumulative rates.

## Abbreviations

CARS-CoV: contagious acute respiratory syndrome coronavirus
CNHC: China's National Health Commission
GBNC: Google Books Ngram Corpus
GTI: Google Trends Index
HARS-CoV: *Han* acute respiratory syndrome coronavirus
HCoV: human coronavirus
HCoV-19: human coronavirus 2019
ICNV: International Committee on Nomenclature of Viruses
ICTV: International Committee on Virus Taxonomy
ICTV-CSG: Coronavirus Study Group of the International Committee on Taxonomy of Viruses
IHR: International Health Regulations
MERS-CoV: Middle East respiratory syndrome–related coronavirus
NCP: novel coronavirus pneumonia
PARS-CoV: pneumonia acute respiratory syndrome coronavirus
PHEIC: Public Health Emergency of International Concern
SARS-CoV: severe acute respiratory syndrome–related coronavirus
TARS-CoV: transmissible acute respiratory syndrome coronavirus
UNESCO: United Nations Educational, Scientific and Cultural Organization
WH-Human-1 coronavirus: Wuhan-Human-1 coronavirus
WHO: World Health Organization
WoS: Web of Science
2019-nCoV: novel coronavirus
