# Supplementary material for: The COVID-19 Infodemic: Infodemiology Study Analyzing Stigmatizing Search Terms
Source: J Med Internet Res. 2020 Nov 16;22(11):e22639. doi: 10.2196/22639 (PMC7674145; doi:10.2196/22639)
Supplement: Multimedia Appendix 1 [file jmir_v22i11e22639_app1.docx]

**Multimedia Appendix 1** The transdisciplinary nature of infodemiology.

**The transdisciplinary nature of infodemiology**

Scientometric analysis promises to articulate the unfolding chronological picture of this branch [1]. In 2002, Gunther Eysenbach first coined the term “infodemiology” – a novel transdisciplinary branch to unravel the complex propagation patterns of the infodemic, along with the term “infoveillance” – the type of syndromic surveillance that utilizes the online contents [2–6]. Nowadays, infodemiological methodology play an important role of addressing pressing issues in public health, including the epidemiology of infodemic [7].

**Figure A1** shows the distributions of records of publications on infodemiology in WoS and PubMed. This distribution indicates that infodemiology is expected to be a full-fledged scientific branch. **Figure** **A2** shows a dual-map overlay of publications on infodemiology in the WoS, with citation links bundled by z-scores. The major trajectory bundles in the dual-map overlay of publications also appear in its followers’ dual-map overlay visualization. As we can see, the major connections take place in the three dominant disciplinary domains. This result strongly suggests that infodemiology is a transdisciplinary branch.

**Figure A1**. Distributions of records of publications on infodemiology in WoS and PubMed (from 2000 to 2020).


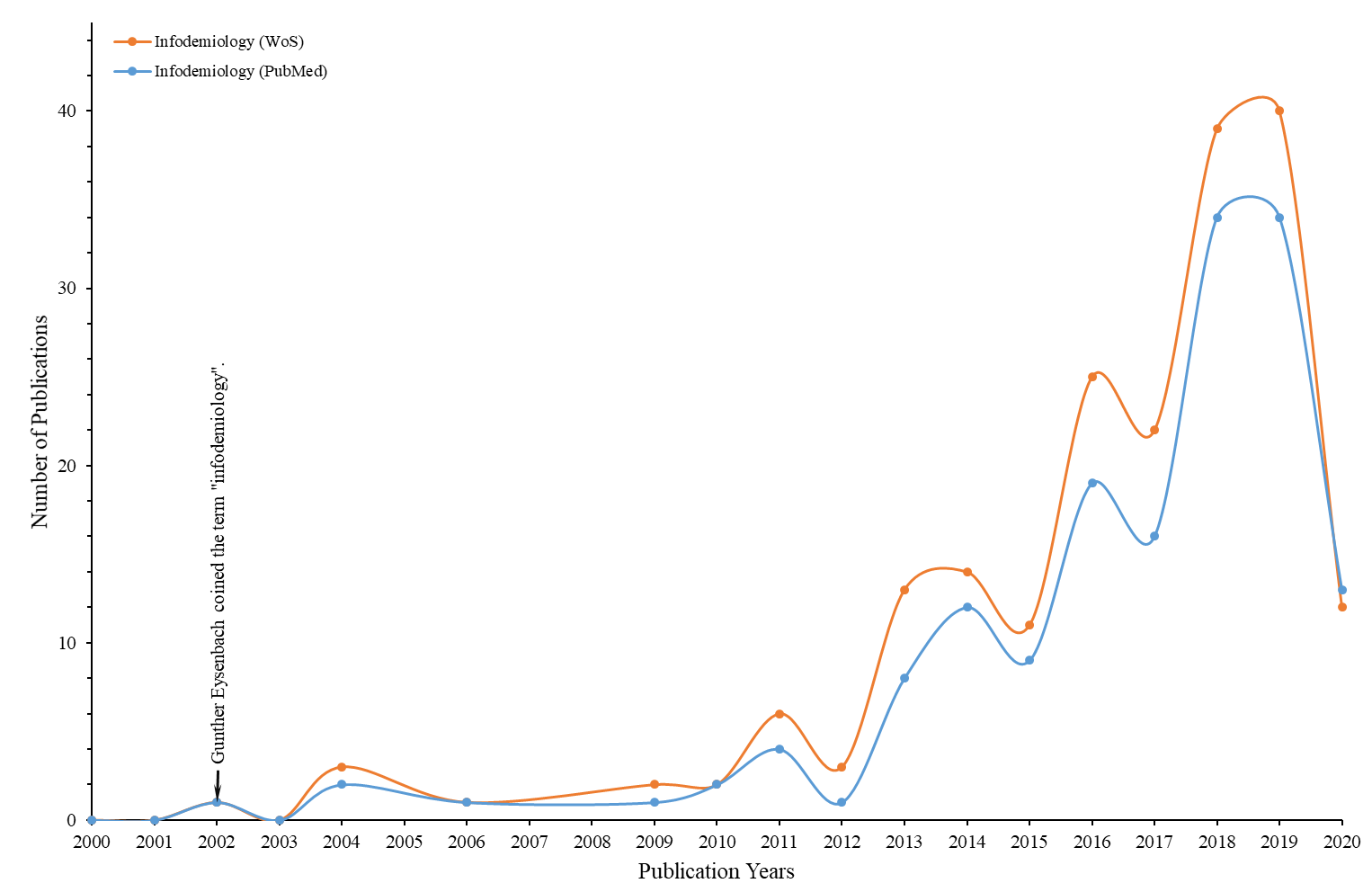


**Figure A2**. A dual-map overlay of publications on infodemiology in the WoS (from 2000 to 2020).


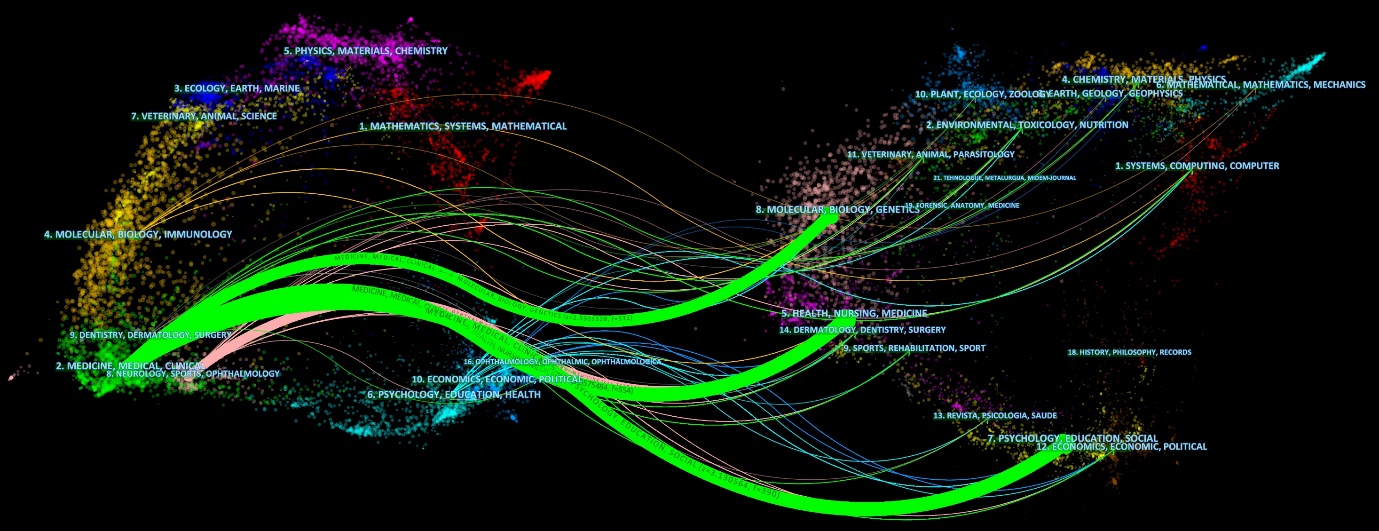


**References:**

1. Hu Z, Cui Y, Zhang J, Eviston-Putsch J. Shalosh B. Ekhad: a computer credit for mathematicians. Scientometrics 2020 Jan 22;122(1):71–97. [doi: 10.1007/s11192-019-03305-7]

2. Eysenbach G. Infodemiology and Infoveillance: Framework for an Emerging Set of Public Health Informatics Methods to Analyze Search, Communication and Publication Behavior on the Internet. J Med Internet Res 2009 Mar 27;11(1):e11. [doi: 10.2196/jmir.1157]

3. Eysenbach G. Infodemiology and Infoveillance Tracking Online Health Information and Cyberbehavior for Public Health. Am J Prev Med 2011 May;40(5):S154–S158. [doi: 10.1016/j.amepre.2011.02.006]

4. Horvitz E, Mulligan D. Data, privacy, and the greater good. Science 2015 Jul 17;349(6245):253–255. [doi: 10.1126/science.aac4520]

5. Editorial. The truth is out there, somewhere. Lancet 2020 Aug;396(10247):291. [doi: 10.1016/S0140-6736(20)31678-0]

6. Editorial. The COVID-19 infodemic. Lancet Infect Dis 2020 Aug;20(8):875. [doi: 10.1016/S1473-3099(20)30565-X]

7. Eysenbach G. Infodemiology: the epidemiology of (mis)information. Am J Med 2002 Dec;113(9):763–765. [doi: 10.1016/S0002-9343(02)01473-0]
