# Supplementary material for: The COVID-19 Infodemic: Infodemiology Study Analyzing Stigmatizing Search Terms
Source: J Med Internet Res. 2020 Nov 16;22(11):e22639. doi: 10.2196/22639 (PMC7674145; doi:10.2196/22639)

**Multimedia Appendix 2** Diachronic discourse analysis and Scientometric analysis.

**Diachronic discourse analysis**

The GBNC facsimiles the word frequency of *coronavirus*, *coronaviruses*, *Coronaviridae* and *Nidovirales* in English corpus from 1963 to 2008, respectively (**Figure A3**). After the initial description of coronaviruses in 1968, there was a mild increase in the numbers of printed books dealing with them, followed by several peaks, after several human coronavirus epidemics: SARS-CoV in 2002-2003, HCoV-NL63 in 2004, and HCoV-HKU1 in 2005. In 1971, *Coronavirus* was officially approved by ICNV. The terms *Coronaviridae*, *Nidovirales* and *Coronavirinae* were officially approved by ICTV in 1975, 1996, 2009, respectively.

**Figure A3**. Diachronic discourse of *coronavirus*, *coronaviruses*, *Coronaviridae* and *Nidovirales* in English corpus.


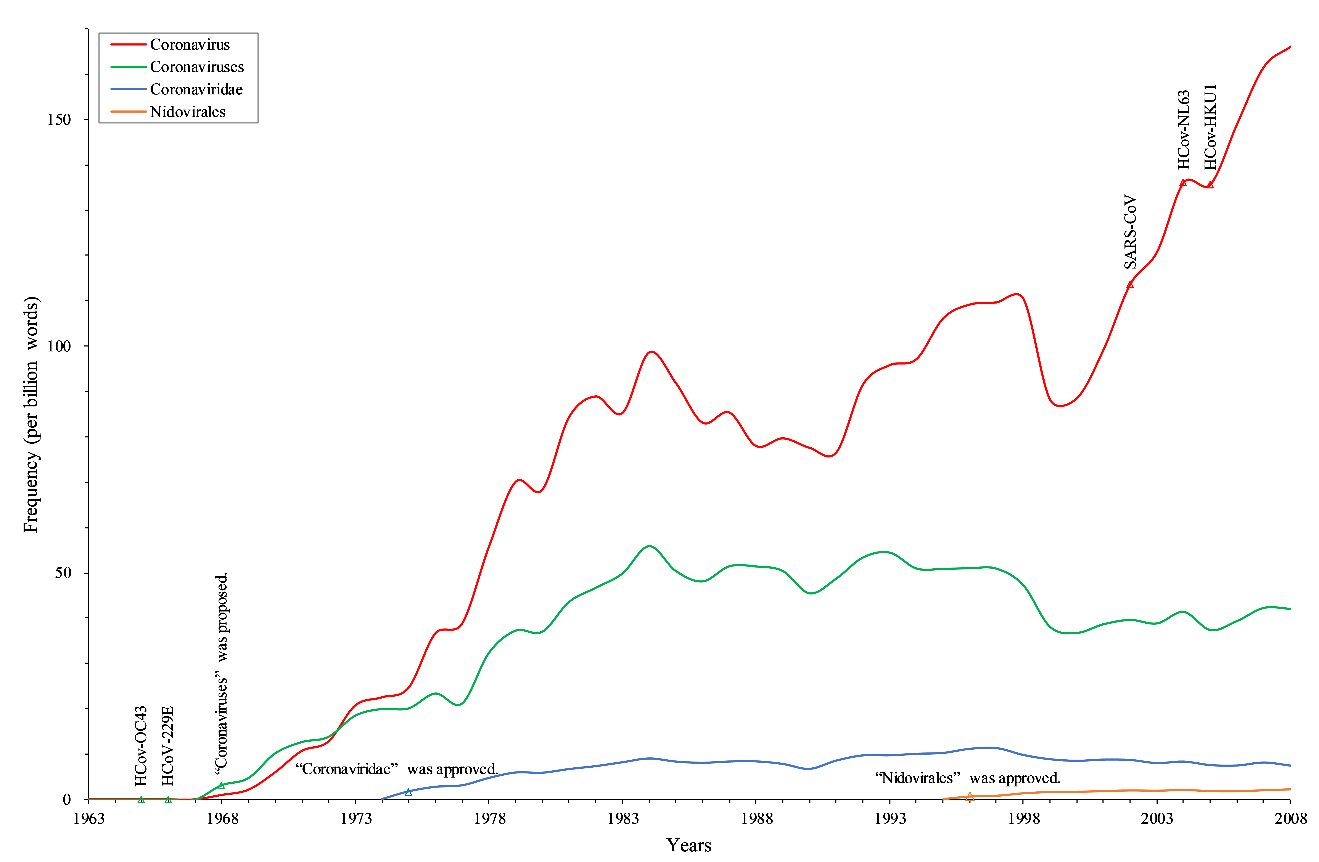


**Scientometric analysis**

Coronaviruses are positive-sense single-stranded RNA viruses found in a wide variety of animals, as well as in humans. Seven strains of respiratory coronaviruses known to infect human-to-human: HCoV-OC43 (1965), HCoV-229E (1966), SARS-CoV (2002), HCoV-NL63 (2004), HCoV-HKU1 (2005), MERS-CoV (2012) and SARS-CoV-2 (2019)(**Figure A4**). WHO declared the 2019-nCoV outbreak a public health emergency of international concern (PHEIC) on 30 January, 2020. This is the 6^th^ time WHO has declared a PHEIC since the International Health Regulations (IHR) came into force in 2005. Before that, there have been five global health emergencies since such declaration was formalized: swine flu (2009), polio (2014), Ebola (2014 then again in 2019), and Zika (2016).

**Figure A4.** Distributions of records of publications on *coronavirus* in WoS and PubMed.


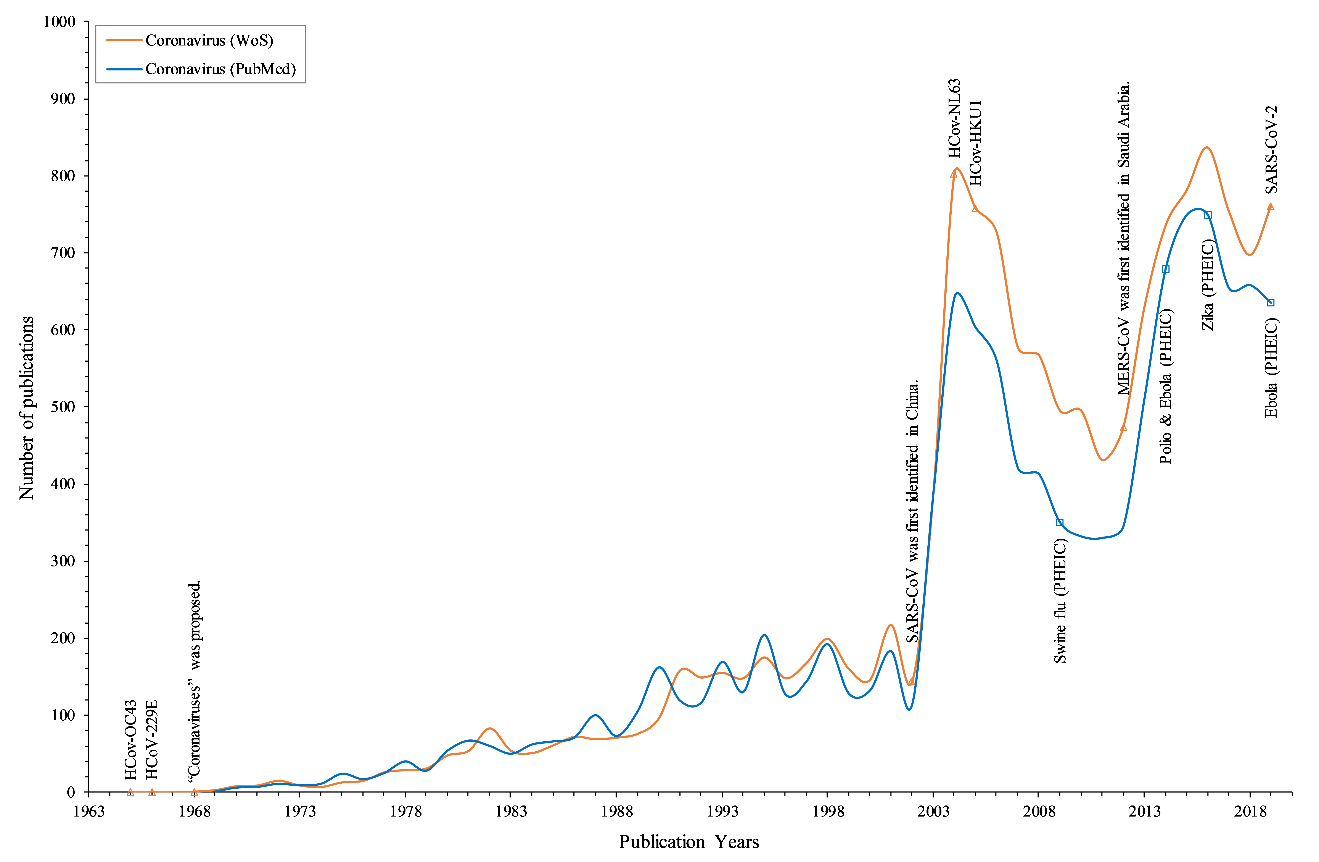

Supplement: Multimedia Appendix 2 [file jmir_v22i11e22639_app2.docx]
