# Supplementary material for: The COVID-19 Infodemic: Infodemiology Study Analyzing Stigmatizing Search Terms
Source: J Med Internet Res. 2020 Nov 16;22(11):e22639. doi: 10.2196/22639 (PMC7674145; doi:10.2196/22639)
Supplement: Multimedia Appendix 3 [file jmir_v22i11e22639_app3.docx]

**Multimedia Appendix 3** Code schemes.

**Code scheme of multifarious naming practices**

A defined itemized code scheme of crowd behavior in daily communication is paramount for our understanding of the unbiased, anonymized sample of Google Trends data available. The initial search candidates included ‘COVID-19’, ‘2019-nCoV’, ‘SARS-CoV-2’, ‘Wuhan pneumonia’, ‘Wuhan virus’, ‘Wuhan coronavirus’, ‘Wuhan corona’, ‘China pneumonia’, ‘China virus’, ‘China coronavirus’, ‘China corona’, ‘Chinese pneumonia’, ‘Chinese virus’, ‘Chinese corona’, ‘novel pneumonia’, ‘novel corona’, ‘novel coronavirus’ and ‘Novel Coronavirus Pneumonia’ (**Table A1**). Then, we filtered candidates of subjective searches in daily communication by three main principles: (i) Search interest on the top of the ranks; (ii) Be formal and complete in spelling; (iii) As much as possible consistent with global crowd participant. Finally, these candidates fit the inclusion criteria were divided into three groups: the official portfolio (‘COVID-19’, ‘2019-nCoV’, ‘SARS-CoV-2’, ‘Novel Coronavirus Pneumonia’), the stigmatized agents (‘Wuhan coronavirus’, ‘China coronavirus’, ‘Chinese coronavirus’, ‘Wuhan pneumonia’, ‘China pneumonia’) and the other counterparts (‘novel pneumonia’, ‘novel coronavirus’).

The final code scheme allows us to measure the relative search interest in the COVID-19 infodemic across Google search and from around the globe. The unbiased and normalized search interest over time provides a unique lens into what netizens were inherently curious about and how they reacted to the COVID-19 epidemic in the real world.

**Table A1.** Code scheme of multifarious naming practices for Google Trends (12/30/2019 - 7/15/2020, Worldwide).

| **Keywords** | **Related Queries^a^** | | | |
| --- | --- | --- | --- | --- |
|  | **Top** | **Percent increase (%)** | **Rising** | **Tremendous increase** |
| **COVID-19** | covid | 100 | covid | Breakout |
|  | coronavirus covid-19 | 89 | coronavirus covid-19 | Breakout |
|  | covid 19 | 65 | covid 19 | Breakout |
|  | covid-19 cases | 61 | covid-19 cases | Breakout |
|  | covid-19 news | 53 | covid-19 news | Breakout |
|  | covid-19 virus | 32 | covid-19 virus | Breakout |
|  | covid-19 india | 29 | covid-19 india | Breakout |
|  | covid-19 update | 26 | covid-19 update | Breakout |
|  | covid-19 symptoms | 23 | covid-19 symptoms | Breakout |
|  | what is covid-19 | 21 | what is covid-19 | Breakout |
|  | covid-19 world | 20 | covid-19 world | Breakout |
|  | covid-19 updates | 20 | covid-19 updates | Breakout |
|  | covid-19 tracker | 19 | covid-19 tracker | Breakout |
|  | covid-19 map | 19 | covid-19 map | Breakout |
|  | covid-19 test | 18 | covid-19 test | Breakout |
|  | covid-19 live | 18 | covid-19 live | Breakout |
|  | covid-19 deaths | 17 | covid-19 deaths | Breakout |
|  | covid-19 us | 17 | covid-19 us | Breakout |
|  | covid-19 who | 15 | covid-19 who | Breakout |
|  | covid-19 testing | 15 | covid-19 testing | Breakout |
|  | covid-19 usa | 13 | covid-19 usa | Breakout |
|  | covid-19 today | 13 | covid-19 today | Breakout |
|  | canada covid-19 | 13 | canada covid-19 | Breakout |
|  | china covid-19 | 11 | china covid-19 | Breakout |
|  | covid-19 pandemic | 11 | covid-19 pandemic | Breakout |
| **2019-nCoV** | coronavirus 2019-ncov | 100 | coronavirus 2019-ncov | Breakout |
|  | coronavirus | 95 | coronavirus | Breakout |
|  | 2019-ncov virus | 34 | 2019-ncov virus | Breakout |
|  | 2019-ncov global cases | 22 | 2019-ncov global cases | Breakout |
|  | ncov | 22 | ncov | Breakout |
|  | коронавирус 2019-ncov | 20 | коронавирус 2019-ncov | Breakout |
|  | 2019-ncov map | 20 | 2019-ncov map | Breakout |
|  | коронавирус | 20 | коронавирус | Breakout |
|  | wuhan | 17 | wuhan | Breakout |
|  | corona virus | 15 | corona virus | Breakout |
|  | 2019 ncov | 13 | 2019 ncov | Breakout |
|  | koronawirus | 13 | koronawirus | Breakout |
|  | koronawirus 2019-ncov | 12 | koronawirus 2019-ncov | Breakout |
|  | sars | 12 | sars | Breakout |
|  | 2019-ncov symptoms | 12 | 2019-ncov symptoms | Breakout |
|  | cdc | 11 | cdc | Breakout |
|  | who 2019-ncov | 11 | who 2019-ncov | Breakout |
|  | who | 11 | who | Breakout |
|  | 2019-ncov global cases by johns hopkins | 10 | 2019-ncov global cases by johns hopkins | Breakout |
|  | 2019-ncov wiki | 9 | 2019-ncov wiki | Breakout |
|  | novel coronavirus | 9 | novel coronavirus | Breakout |
|  | 2019-ncov news | 9 | 2019-ncov news | Breakout |
|  | novel coronavirus 2019-ncov | 8 | novel coronavirus 2019-ncov | Breakout |
|  | coronavirus map | 8 | coronavirus map | Breakout |
|  | 2019-ncov global cases by johns hopkins csse | 7 | 2019-ncov global cases by johns hopkins csse | Breakout |
| **SARS-CoV-2** | coronavirus sars-cov-2 | 100 | coronavirus sars-cov-2 | Breakout |
|  | covid-19 | 98 | covid-19 | Breakout |
|  | covid-19 sars-cov-2 | 98 | covid-19 sars-cov-2 | Breakout |
|  | covid | 88 | covid | Breakout |
|  | virus sars-cov-2 | 61 | virus sars-cov-2 | Breakout |
|  | sars | 60 | sars | Breakout |
|  | sars-cov-2 covid 19 | 52 | sars-cov-2 covid 19 | Breakout |
|  | covid 19 | 48 | covid 19 | Breakout |
|  | sars-cov-2 test | 43 | sars-cov-2 test | Breakout |
|  | what is sars-cov-2 | 24 | what is sars-cov-2 | Breakout |
|  | sars-cov-2 vs covid-19 | 17 | sars-cov-2 vs covid-19 | Breakout |
|  | sars cov 2 | 17 | sars cov 2 | Breakout |
|  | sars-cov-2 igg | 16 | sars-cov-2 igg | Breakout |
|  | sars-cov-2 rna | 14 | sars-cov-2 rna | Breakout |
|  | covid19 | 12 | covid19 | Breakout |
|  | que es sars-cov-2 | 11 | que es sars-cov-2 | Breakout |
|  | sars-cov-2 pcr | 11 | sars-cov-2 pcr | Breakout |
|  | sars-cov | 11 | sars-cov | Breakout |
|  | sars virus | 10 | sars virus | Breakout |
|  | sars-cov-2 origin | 10 | sars-cov-2 origin | Breakout |
|  | sars-cov-2 antibody test | 9 | sars-cov-2 antibody test | Breakout |
|  | sars-cov-2 vaccine | 9 | sars-cov-2 vaccine | Breakout |
|  | sars-cov-1 | 8 | sars-cov-1 | Breakout |
|  | que es el sars-cov-2 | 8 | que es el sars-cov-2 | Breakout |
|  | sars-cov-2 wiki | 7 | sars-cov-2 wiki | Breakout |
| **Wuhan pneumonia** | wuhan virus | 100 | wuhan virus | Breakout |
|  | wuhan coronavirus | 86 | wuhan coronavirus | Breakout |
|  | wuhan pneumonia coronavirus | 80 | wuhan pneumonia coronavirus | Breakout |
|  | wuhan pneumonia symptoms | 62 | wuhan pneumonia symptoms | Breakout |
|  | china wuhan pneumonia | 58 | china wuhan pneumonia | Breakout |
|  | pneumonia symptoms | 56 | pneumonia symptoms | Breakout |
|  | singapore wuhan pneumonia | 54 | singapore wuhan pneumonia | Breakout |
|  | symptoms of wuhan pneumonia | 27 | symptoms of wuhan pneumonia | Breakout |
|  | wuhan pneumonia news | 24 | wuhan pneumonia news | Breakout |
|  | moh wuhan pneumonia | 22 | moh wuhan pneumonia | Breakout |
|  | symptoms of pneumonia | 22 | symptoms of pneumonia | Breakout |
|  | wuhan virus symptoms | 22 | wuhan virus symptoms | Breakout |
|  | wuhan pneumonia hong kong | 21 | wuhan pneumonia hong kong | Breakout |
|  | wuhan pneumonia wiki | 20 | wuhan pneumonia wiki | Breakout |
|  | wuhan pneumonia outbreak | 19 | wuhan pneumonia outbreak | Breakout |
|  | wuhan pneumonia update | 18 | wuhan pneumonia update | Breakout |
|  | wuhan virus singapore | 15 | wuhan virus singapore | Breakout |
|  | 武漢 肺炎 | 13 | 武漢 肺炎 | Breakout |
|  | wuhan coronavirus symptoms | 13 | wuhan coronavirus symptoms | Breakout |
|  | wuhan pneumonia death | 13 | wuhan pneumonia death | Breakout |
|  | 武汉 肺炎 | 12 | 武汉 肺炎 | Breakout |
|  | wuhan pneumonia hk | 10 | wuhan pneumonia hk | Breakout |
|  | symptoms of wuhan coronavirus | 9 | symptoms of wuhan coronavirus | Breakout |
|  | wuhan pneumonia taiwan | 7 | wuhan pneumonia taiwan | Breakout |
|  | 武汉 肺炎 英文 | 5 | 武汉 肺炎 英文 | Breakout |
| **Wuhan virus** | corona wuhan virus | 100 | corona wuhan virus | Breakout |
|  | corona virus | 100 | corona virus | Breakout |
|  | virus corona wuhan | 97 | virus corona wuhan | Breakout |
|  | singapore wuhan | 77 | singapore wuhan | Breakout |
|  | wuhan singapore virus | 77 | wuhan singapore virus | Breakout |
|  | singapore | 76 | singapore | Breakout |
|  | wuhan coronavirus virus | 76 | wuhan coronavirus virus | Breakout |
|  | wuhan coronavirus | 75 | wuhan coronavirus | Breakout |
|  | wuhan virus coronavirus | 75 | wuhan virus coronavirus | Breakout |
|  | china virus | 65 | china virus | Breakout |
|  | china wuhan virus | 64 | china wuhan virus | Breakout |
|  | wuhan china | 64 | wuhan china | Breakout |
|  | wuhan virus china | 63 | wuhan virus china | Breakout |
|  | china | 62 | china | Breakout |
|  | the wuhan virus | 42 | the wuhan virus | Breakout |
|  | wuhan virus update | 41 | wuhan virus update | Breakout |
|  | wuhan news | 31 | wuhan news | Breakout |
|  | wuhan virus news | 31 | wuhan virus news | Breakout |
|  | latest wuhan virus | 25 | latest wuhan virus | Breakout |
|  | wuhan china corona virus | 22 | wuhan china corona virus | Breakout |
|  | corona virus china | 22 | corona virus china | Breakout |
|  | corona virus in wuhan | 22 | corona virus in wuhan | Breakout |
|  | wuhan virus symptoms | 20 | wuhan virus symptoms | Breakout |
|  | coronavirus china | 19 | coronavirus china | Breakout |
|  | wuhan china virus coronavirus | 19 | wuhan china virus coronavirus | Breakout |
| **Wuhan coronavirus** | coronavirus china wuhan | 100 | coronavirus china wuhan | Breakout |
|  | china coronavirus | 99 | china coronavirus | Breakout |
|  | china wuhan | 99 | china wuhan | Breakout |
|  | china | 99 | china | Breakout |
|  | coronavirus in wuhan | 74 | coronavirus in wuhan | Breakout |
|  | virus | 62 | virus | Breakout |
|  | wuhan virus | 62 | wuhan virus | Breakout |
|  | wuhan virus coronavirus | 59 | wuhan virus coronavirus | Breakout |
|  | the wuhan coronavirus | 37 | the wuhan coronavirus | Breakout |
|  | wuhan corona | 33 | wuhan corona | Breakout |
|  | coronavirus de wuhan | 30 | coronavirus de wuhan | Breakout |
|  | coronavirus in china | 27 | coronavirus in china | Breakout |
|  | coronavirus in wuhan china | 26 | coronavirus in wuhan china | Breakout |
|  | corona virus wuhan | 21 | corona virus wuhan | Breakout |
|  | corona virus | 21 | corona virus | Breakout |
|  | coronavirus cases | 21 | coronavirus cases | Breakout |
|  | wuhan coronavirus cases | 20 | wuhan coronavirus cases | Breakout |
|  | coronavirus news | 19 | coronavirus news | Breakout |
|  | coronavirus wuhan update | 19 | coronavirus wuhan update | Breakout |
|  | wuhan news | 19 | wuhan news | Breakout |
|  | wuhan coronavirus news | 18 | wuhan coronavirus news | Breakout |
|  | coronavirus wuhan symptoms | 18 | coronavirus wuhan symptoms | Breakout |
|  | coronavirus symptoms | 18 | coronavirus symptoms | Breakout |
|  | coronavirus update | 18 | coronavirus update | Breakout |
|  | symptoms | 17 | symptoms | Breakout |
| **Wuhan corona** | corona virus | 100 | corona virus | Breakout |
|  | virus wuhan | 100 | virus wuhan | Breakout |
|  | corona wuhan virus | 100 | corona wuhan virus | Breakout |
|  | virus corona wuhan | 100 | virus corona wuhan | Breakout |
|  | virus | 99 | virus | Breakout |
|  | wuhan coronavirus | 43 | wuhan coronavirus | Breakout |
|  | wuhan china | 41 | wuhan china | Breakout |
|  | china wuhan corona | 40 | china wuhan corona | Breakout |
|  | china | 40 | china | Breakout |
|  | corona in wuhan | 40 | corona in wuhan | Breakout |
|  | corona china | 40 | corona china | Breakout |
|  | virus corona china wuhan | 25 | virus corona china wuhan | Breakout |
|  | corona virus china | 25 | corona virus china | Breakout |
|  | corona virus in wuhan | 24 | corona virus in wuhan | Breakout |
|  | virus wuhan china | 23 | virus wuhan china | Breakout |
|  | corona di wuhan | 16 | corona di wuhan | Breakout |
|  | corona in china | 13 | corona in china | Breakout |
|  | china coronavirus wuhan | 13 | china coronavirus wuhan | Breakout |
|  | china coronavirus | 13 | china coronavirus | Breakout |
|  | wuhan corona cases | 9 | wuhan corona cases | Breakout |
|  | virus corona di wuhan | 9 | virus corona di wuhan | Breakout |
|  | corona virus in china | 8 | corona virus in china | Breakout |
|  | wuhan news | 8 | wuhan news | Breakout |
|  | corona news | 8 | corona news | Breakout |
|  | corona update | 8 | corona update | Breakout |
| **China pneumonia** | pneumonia in china | 100 | coronavirus china pneumonia | Breakout |
|  | coronavirus china pneumonia | 91 | coronavirus | Breakout |
|  | coronavirus | 89 | china coronavirus | Breakout |
|  | china coronavirus | 87 | wuhan pneumonia | Breakout |
|  | china virus | 50 | wuhan | Breakout |
|  | wuhan pneumonia | 47 | corona virus china | Breakout |
|  | wuhan | 44 | 2019-20 china pneumonia outbreak | Breakout |
|  | china pneumonia outbreak | 30 | a novel coronavirus from patients with pneumonia in china | Breakout |
|  | corona virus china | 12 | mysterious pneumonia in china | Breakout |
|  | 2019-20 china pneumonia outbreak | 5 | 武汉 肺炎 | Breakout |
|  | a novel coronavirus from patients with pneumonia in china | 2 | china virus | 80% |
|  | mysterious pneumonia in china | 2 |  |  |
|  | 武汉 肺炎 | 2 |  |  |
| **China virus** | corona china | 100 | corona china | Breakout |
|  | china corona virus | 99 | china corona virus | Breakout |
|  | corona virus | 98 | corona virus | Breakout |
|  | virus corona | 97 | virus corona | Breakout |
|  | virus in china | 74 | corona virus in china | Breakout |
|  | china virus coronavirus | 49 | china virus 2020 | Breakout |
|  | china coronavirus | 48 | china virus wuhan | Breakout |
|  | coronavirus china | 48 | china virus update | Breakout |
|  | corona virus in china | 34 | china virus death | Breakout |
|  | coronavirus in china | 18 | corona virus news china | Breakout |
|  | new virus china | 17 | corona virus news | Breakout |
|  | new virus | 17 | china corona virus cases | Breakout |
|  | virus from china | 16 | corona virus cases | Breakout |
|  | the virus in china | 15 | india corona virus | Breakout |
|  | news china virus | 15 | corona virus update china | Breakout |
|  | china news | 14 | corona virus update | Breakout |
|  | china virus 2020 | 13 | italy | Breakout |
|  | wuhan china | 13 | china virus latest | Breakout |
|  | china virus wuhan | 13 | china virus trump | Breakout |
|  | wuhan virus | 13 | trump china virus | Breakout |
|  | wuhan | 13 | china virus symptoms | Breakout |
|  | virus en china | 11 | china virus deaths | Breakout |
|  | virus de china | 11 | who china virus | Breakout |
|  | new virus in china | 11 | who | Breakout |
|  | china virus update | 9 | virus in china 2020 | Breakout |
| **China coronavirus** | coronavirus in china | 100 | coronavirus in china | Breakout |
|  | china virus | 34 | china virus | Breakout |
|  | corona china | 34 | corona china | Breakout |
|  | coronavirus en china | 31 | coronavirus en china | Breakout |
|  | china coronavirus cases | 28 | china coronavirus cases | Breakout |
|  | coronavirus cases | 28 | coronavirus cases | Breakout |
|  | china news coronavirus | 25 | china news coronavirus | Breakout |
|  | coronavirus news | 25 | coronavirus news | Breakout |
|  | china news | 25 | china news | Breakout |
|  | coronavirus update china | 23 | coronavirus update china | Breakout |
|  | coronavirus update | 23 | coronavirus update | Breakout |
|  | corona virus china | 19 | corona virus china | Breakout |
|  | corona virus | 19 | corona virus | Breakout |
|  | coronavirus india | 19 | coronavirus india | Breakout |
|  | india coronavirus | 19 | india coronavirus | Breakout |
|  | india china | 19 | india china | Breakout |
|  | china coronavirus wuhan | 18 | china coronavirus wuhan | Breakout |
|  | wuhan coronavirus | 18 | wuhan coronavirus | Breakout |
|  | wuhan china | 18 | wuhan china | Breakout |
|  | wuhan | 17 | wuhan | Breakout |
|  | italy coronavirus | 17 | italy coronavirus | Breakout |
|  | italy | 16 | italy | Breakout |
|  | china coronavirus death | 16 | china coronavirus death | Breakout |
|  | us coronavirus | 15 | us coronavirus | Breakout |
|  | coronavirus cases in china | 14 | coronavirus cases in china | Breakout |
| **China corona** | virus china | 100 | virus china | Breakout |
|  | china virus corona | 97 | china virus corona | Breakout |
|  | corona virus | 96 | corona virus | Breakout |
|  | corona in china | 72 | coronavirus china | Breakout |
|  | coronavirus china | 51 | corona virus in china | Breakout |
|  | corona virus in china | 37 | virus in china | Breakout |
|  | virus in china | 37 | coronavirus in china | Breakout |
|  | coronavirus in china | 19 | corona cases | Breakout |
|  | corona cases | 18 | china corona cases | Breakout |
|  | china corona cases | 18 | china update | Breakout |
|  | india china | 15 | corona cases in china | Breakout |
|  | corona india | 15 | corona update china | Breakout |
|  | india | 15 | corona update | Breakout |
|  | corona china news | 14 | corona in india | Breakout |
|  | corona news | 14 | italy | Breakout |
|  | china news | 14 | corona italy | Breakout |
|  | china update | 12 | china corona death | Breakout |
|  | corona cases in china | 12 | corona na china | Breakout |
|  | corona update china | 12 | wuhan | Breakout |
|  | corona update | 11 | wuhan china | Breakout |
|  | corona in india | 9 | wuhan china corona | Breakout |
|  | italy | 9 | corona virus news | Breakout |
|  | corona italy | 9 | corona virus news china | Breakout |
|  | china corona death | 8 | china coronavirus cases | Breakout |
|  | corona na china | 8 | corona world | Breakout |
| **Chinese pneumonia** | pneumonia in chinese | 100 | wuhan pneumonia | Breakout |
|  | chinese virus | 35 | chinese virus | 150% |
|  | wuhan pneumonia | 27 |  |  |
| **Chinese virus** | corona chinese virus | 100 | corona chinese virus | Breakout |
|  | corona virus | 99 | corona virus | Breakout |
|  | virus in chinese | 63 | chinese coronavirus | Breakout |
|  | the chinese virus | 63 | trump chinese virus | Breakout |
|  | chinese coronavirus | 54 | trump | Breakout |
|  | china | 40 | corona virus in chinese | Breakout |
|  | china virus | 39 | chinese virus 2020 | Breakout |
|  | trump chinese virus | 37 | wuhan virus | Breakout |
|  | trump | 37 | wuhan | Breakout |
|  | corona virus in chinese | 27 | chinese wuhan virus | Breakout |
|  | new chinese virus | 21 | corona virus china | Breakout |
|  | chinese virus 2020 | 20 | coronavirus china | Breakout |
|  | wuhan virus | 18 | chinese virus symptoms | Breakout |
|  | wuhan | 18 | trump the chinese virus | Breakout |
|  | chinese wuhan virus | 18 | wuhan virus in chinese | Breakout |
|  | corona virus china | 16 | chinese virus in us | Breakout |
|  | chinese virus news | 16 | donald trump | Breakout |
|  | virus in china | 16 | donald trump chinese virus | Breakout |
|  | coronavirus china | 12 | italy | Breakout |
|  | chinese virus symptoms | 10 | corona virus news | Breakout |
|  | what is chinese virus | 10 | chinese virus deaths | Breakout |
|  | who chinese virus | 10 | sars | Breakout |
|  | chinese flu | 8 | chinese lab virus | Breakout |
|  | chinese flu virus | 7 | chinese virus latest | Breakout |
|  | chinese virus uk | 7 | coronavirus symptoms | Breakout |
| **Chinese coronavirus** | coronavirus in chinese | 100 | coronavirus in chinese | Breakout |
|  | coronavirus china chinese | 57 | virus | Breakout |
|  | coronavirus china | 56 | chinese virus | Breakout |
|  | china | 56 | chinese corona | Breakout |
|  | virus | 49 | coronavirus in china | Breakout |
|  | chinese virus | 46 | corona virus | Breakout |
|  | chinese corona | 34 | chinese coronavirus news | Breakout |
|  | coronavirus in china | 23 | chinese news | Breakout |
|  | corona virus | 21 | coronavirus chinese food | Breakout |
|  | chinese coronavirus news | 21 | chinese coronavirus cases | Breakout |
|  | chinese news | 21 | coronavirus news | Breakout |
|  | coronavirus chinese food | 20 | chinese food | Breakout |
|  | chinese coronavirus cases | 20 | coronavirus cases | Breakout |
|  | coronavirus news | 20 | us coronavirus | Breakout |
|  | chinese food | 20 | chinese coronavirus update | Breakout |
|  | coronavirus cases | 19 | coronavirus update | Breakout |
|  | us coronavirus | 18 | wuhan coronavirus | Breakout |
|  | chinese coronavirus update | 15 | wuhan | Breakout |
|  | coronavirus update | 15 | who | Breakout |
|  | wuhan coronavirus | 15 | who coronavirus | Breakout |
|  | wuhan | 14 | chinese doctor | Breakout |
|  | who | 14 | thank you coronavirus helpers | Breakout |
|  | who coronavirus | 14 | chinese doctor coronavirus | Breakout |
|  | chinese doctor | 14 | what is coronavirus | Breakout |
|  | thank you coronavirus helpers | 13 | coronavirus italy | Breakout |
| **Chinese corona** | chinese corona virus | 100 | chinese corona virus | Breakout |
|  | chinese virus | 99 | chinese virus | Breakout |
|  | virus corona | 99 | virus corona | Breakout |
|  | corona in chinese | 53 | chinese coronavirus | Breakout |
|  | chinese coronavirus | 40 | corona virus in chinese | Breakout |
|  | corona virus in chinese | 31 | corona virus china | Breakout |
|  | china | 30 | coronavirus china | Breakout |
|  | corona virus china | 18 | corona cases | Breakout |
|  | coronavirus china | 10 | chinese corona cases | Breakout |
|  | corona cases | 10 | wuhan | Breakout |
|  | chinese corona cases | 9 | corona update | Breakout |
|  | chinese food | 8 | corona virus news | Breakout |
|  | wuhan | 8 | new chinese virus | Breakout |
|  | corona update | 7 | corona in india | Breakout |
|  | corona virus news | 6 | chinese corona deaths | Breakout |
|  | new chinese virus | 6 | trump chinese virus | Breakout |
|  | corona in india | 5 | corona virus update | Breakout |
|  | corona meaning | 5 | corona symptoms | Breakout |
|  | chinese corona deaths | 4 | chinese new year | Breakout |
|  | trump chinese virus | 4 | corona virus india | Breakout |
|  | corona virus update | 4 | corona vaccine | Breakout |
|  | chinese restaurant | 4 | chinese lab corona | Breakout |
|  | corona symptoms | 3 | chinese flu | Breakout |
|  | chinese new year | 3 | corona virus symptoms | Breakout |
|  | corona virus india | 3 | corona virus usa | Breakout |
| **novel pneumonia** | novel coronavirus pneumonia | 100 | novel coronavirus pneumonia | Breakout |
|  | early transmission dynamics in wuhan, china, of novel coronavirus-infected pneumonia | 9 | early transmission dynamics in wuhan, china, of novel coronavirus-infected pneumonia | Breakout |
|  | a novel coronavirus from patients with pneumonia in china, 2019 | 8 | a novel coronavirus from patients with pneumonia in china, 2019 | Breakout |
|  | early transmission dynamics in wuhan, china, of novel coronavirus-infected pneumonia | 6 | early transmission dynamics in wuhan, china, of novel coronavirus-infected pneumonia | Breakout |
|  | a novel coronavirus from patients with pneumonia in china | 3 | a novel coronavirus from patients with pneumonia in china | Breakout |
|  | clinical characteristics of 138 hospitalized patients with 2019 novel ccoronavirus-infected pneumonia in wuhan, china | 3 | clinical characteristics of 138 hospitalized patients with 2019 novel coronavirus-infected pneumonia in wuhan, china | Breakout |
| **novel corona** | novel virus corona | 100 | novel virus corona | Breakout |
|  | corona virus | 100 | corona virus | Breakout |
|  | novel corona virus | 99 | novel corona virus | Breakout |
|  | novel coronavirus | 60 | novel coronavirus | Breakout |
|  | novel meaning | 18 | novel meaning | Breakout |
|  | novel corona meaning | 17 | novel corona meaning | Breakout |
|  | what is novel corona | 17 | corona meaning | Breakout |
|  | corona meaning | 17 | covid | Breakout |
|  | covid | 17 | covid 19 | Breakout |
|  | covid 19 | 14 | what is novel corona virus | Breakout |
|  | what is novel corona virus | 14 | what is corona virus | Breakout |
|  | what is corona virus | 14 | what is coronavirus | Breakout |
|  | what is coronavirus | 11 | what is novel coronavirus | Breakout |
|  | what is novel coronavirus | 10 | why is corona called novel | Breakout |
|  | why is corona called novel | 10 | novel corona virus symptoms | Breakout |
|  | novel corona virus symptoms | 8 | corona virus symptoms | Breakout |
|  | corona virus symptoms | 7 | corona virus india | Breakout |
|  | corona virus india | 7 | novel coronavirus meaning | Breakout |
|  | novel coronavirus meaning | 7 | novel coronavirus symptoms | Breakout |
|  | novel coronavirus symptoms | 7 | why corona virus is called novel | Breakout |
|  | why corona virus is called novel | 7 | symptoms of corona virus | Breakout |
|  | symptoms of corona virus | 5 | symptoms of novel corona virus | Breakout |
|  | symptoms of novel corona virus | 5 | novel corona map | Breakout |
|  | novel corona map | 5 | why coronavirus is called novel | Breakout |
|  | why coronavirus is called novel | 4 | novel corona virus update | Breakout |
| **novel coronavirus** | the novel coronavirus | 100 | the novel coronavirus | Breakout |
|  | novel coronavirus virus | 63 | novel coronavirus virus | Breakout |
|  | novel virus | 61 | novel virus | Breakout |
|  | novel corona | 57 | novel corona | Breakout |
|  | 2019 novel coronavirus | 45 | 2019 novel coronavirus | Breakout |
|  | novel coronavirus meaning | 43 | novel coronavirus meaning | Breakout |
|  | novel meaning | 43 | novel meaning | Breakout |
|  | coronavirus symptoms | 42 | coronavirus symptoms | Breakout |
|  | coronavirus novel symptoms | 41 | coronavirus novel symptoms | Breakout |
|  | what is novel | 39 | what is novel | Breakout |
|  | what is novel coronavirus | 38 | what is novel coronavirus | Breakout |
|  | what is coronavirus | 38 | what is coronavirus | Breakout |
|  | novel corona virus | 37 | novel corona virus | Breakout |
|  | why novel coronavirus | 36 | why novel coronavirus | Breakout |
|  | novel coronavirus covid 19 | 29 | novel coronavirus covid 19 | Breakout |
|  | covid 19 | 28 | covid 19 | Breakout |
|  | why is coronavirus novel | 25 | why is coronavirus novel | Breakout |
|  | who novel coronavirus | 25 | who novel coronavirus | Breakout |
|  | novel coronavirus update | 25 | novel coronavirus update | Breakout |
|  | coronavirus update | 25 | coronavirus update | Breakout |
|  | who | 24 | who | Breakout |
|  | who coronavirus | 23 | who coronavirus | Breakout |
|  | novel coronavirus map | 23 | novel coronavirus map | Breakout |
|  | coronavirus map | 23 | coronavirus map | Breakout |
|  | novel coronavirus china | 23 | novel coronavirus china | Breakout |
| **Novel Coronavirus Pneumonia** | a novel coronavirus from patients with pneumonia in china | 100 | a novel coronavirus from patients with pneumonia in china | Breakout |
|  | a novel coronavirus from patients with pneumonia in china, 2019 | 99 | a novel coronavirus from patients with pneumonia in china, 2019 | Breakout |
|  | ncp | 60 | ncp | Breakout |
| ^a^Top - The most popular search queries. Scoring is on a relative scale where a value of 100 is the most commonly searched query, 50 is a query searched half as often as the most popular query, and so on. Rising - Queries with the biggest increase in search frequency since the last time period. Results marked "Breakout" had a tremendous increase, probably because these queries are new and had few (if any) prior searches. | | | | |

Each taxonomic procedure is often an original creation to search through dusty tomes for comparative morphologic synset and crystallize a pithy neologism for the appropriateness of possible antedating description. Unfortunately, before the antidotes to infodemic – proper names – find their ways into the public mind, debate on interim solutions has been going on in scientific community (**Table A2**). Multifarious portmanteaus of the novel coronavirus have been proposed by scientists around the globe, such as “SARS-CoV-2”, “HCoV-19”, “HARS-CoV”, “PARS-CoV”, “TARS-CoV”, “CARS-CoV”, etc. Of these, “SARS-CoV-2” has the highest search interest, followed by “HCoV-19”. The others did not have enough volumes to measure.

**Table A2.** Code scheme of multifarious naming practices in scientific sphere (12/30/2019 - 7/15/2020, Worldwide).

| **Keywords** | **Related Queries^b^** | | | |
| --- | --- | --- | --- | --- |
|  | **Top** | **Scores** | **Rising** | **Increase** |
| **SARS-CoV-2** | coronavirus sars-cov-2 | 100 | coronavirus sars-cov-2 | Breakout |
|  | covid-19 sars-cov-2 | 100 | covid-19 sars-cov-2 | Breakout |
|  | covid-19 | 98 | covid-19 | Breakout |
|  | covid | 86 | covid | Breakout |
|  | sars-cov-2 virus | 66 | sars-cov-2 virus | Breakout |
|  | sars | 62 | sars | Breakout |
|  | sars-cov-2 covid 19 | 52 | sars-cov-2 covid 19 | Breakout |
|  | covid 19 | 51 | covid 19 | Breakout |
|  | sars-cov-2 test | 41 | sars-cov-2 test | Breakout |
|  | what is sars-cov-2 | 25 | what is sars-cov-2 | Breakout |
|  | sars-cov-2 igg | 21 | sars-cov-2 igg | Breakout |
|  | sars-cov-2 vs covid-19 | 19 | sars-cov-2 vs covid-19 | Breakout |
|  | sars cov 2 | 19 | sars cov 2 | Breakout |
|  | sars-cov-2 rna | 17 | sars-cov-2 rna | Breakout |
|  | sars-cov-2 pcr | 15 | sars-cov-2 pcr | Breakout |
|  | covid19 | 13 | covid19 | Breakout |
|  | sars-cov-2 transmission | 13 | sars-cov-2 transmission | Breakout |
|  | who | 13 | who | Breakout |
|  | que es sars-cov-2 | 13 | que es sars-cov-2 | Breakout |
|  | sars-cov | 12 | sars-cov | Breakout |
|  | sars virus | 11 | sars virus | Breakout |
|  | sars-cov-2 antibody test | 11 | sars-cov-2 antibody test | Breakout |
|  | sars-cov-2 igm | 9 | sars-cov-2 igm | Breakout |
|  | sars-cov-2 spike protein | 8 | sars-cov-2 spike protein | Breakout |
|  | sars-cov-2 meaning | 8 | sars-cov-2 meaning | Breakout |
|  | sars-cov-2 meaning | 8 | sars-cov-2 meaning | Breakout |
| **HCoV-19**^c^ | - | - | - | - |
| **HARS-CoV** | - | - | - | - |
| **PARS-CoV** | - | - | - | - |
| **TARS-CoV** | - | - | - | - |
| **CARS-CoV** | - | - | - | - |
| ^b^Top - The most popular search queries. Scoring is on a relative scale where a value of 100 is the most commonly searched query, 50 is a query searched half as often as the most popular query, and so on. Rising - Queries with the biggest increase in search frequency since the last time period. Results marked "Breakout" had a tremendous increase, probably because these queries are new and had few (if any) prior searches.  ^c^HCoV-19 is known as “Human Coronavirus 2019” or “Han Coronavirus 2019”. ‘Han’ is the abbreviation of Wuhan in Chinese. | | | | |

**Code scheme of combating the COVID-19 infodemic**

**Table A3.** Code scheme of combating the COVID-19 infodemic (1/1/2020 - 7/15/2020, Worldwide).

| **Keywords** | **Related Queries**^d^ | | | |
| --- | --- | --- | --- | --- |
|  | **Top** | **Percent increase (%)** | **Rising** | **Tremendous increase** |
| **infodemic** | infodemic meaning | 100 | infodemic meaning | Breakout |
|  | what is infodemic | 25 | what is infodemic | Breakout |
|  | who infodemic | 22 | who infodemic | Breakout |
|  | infodemic definition | 17 | infodemic definition | Breakout |
|  | infodemic 中文 | 12 | infodemic 中文 | Breakout |
|  | infodemic meaning in hindi | 8 | infodemic meaning in hindi | Breakout |
|  | what is an infodemic | 6 | what is an infodemic | Breakout |
|  | infodemic meaning in bengali | 3 | infodemic meaning in bengali | Breakout |
| **disinfodemic** | - | - | - | - |
| **misinfodemic** | - | - | - | - |
| ^d^Top - The most popular search queries. Scoring is on a relative scale where a value of 100 is the most commonly searched query, 50 is a query searched half as often as the most popular query, and so on. Rising - Queries with the biggest increase in search frequency since the last time period. Results marked "Breakout" had a tremendous increase, probably because these queries are new and had few (if any) prior searches. | | | | |
